# Epidemiology of Dengue Disease in the Philippines (2000–2011): A Systematic Literature Review

**DOI:** 10.1371/journal.pntd.0003027

**Published:** 2014-11-06

**Authors:** Lulu Bravo, Vito G. Roque, Jeremy Brett, Ruby Dizon, Maïna L'Azou

**Affiliations:** 1 National Institutes of Health, University of the Philippines Manila (UPM), Manila, Philippines; 2 National Epidemiology Center, Department of Health, Manila, Philippines; 3 Sanofi-Aventis Pte. Ltd., Singapore; 4 Sanofi Pasteur, Manila, Philippines; 5 Global Epidemiology Department, Sanofi Pasteur, Lyon, France; University of Heidelberg, Germany

## Abstract

This literature analysis describes the available dengue epidemiology data in the Philippines between 2000 and 2011. Of 253 relevant data sources identified, 34, including additional epidemiology data provided by the National Epidemiology Center, Department of Health, Philippines, were reviewed. There were 14 publications in peer reviewed journals, and 17 surveillance reports/sources, which provided variable information from the passive reporting system and show broad trends in dengue incidence, including age group predominance and disease severity. The peer reviewed studies focused on clinical severity of cases, some revealed data on circulating serotypes and genotypes and on the seroepidemiology of dengue including incidence rates for infection and apparent disease. Gaps in the data were identified, and include the absence incidence rates stratified by age, dengue serotype and genotype distribution, disease severity data, sex distribution data, and seroprevalence data.

**Protocol registration:**

PROSPERO CRD42012002292

## Author Summary

Dengue disease is a tropical and subtropical mosquito-borne viral illness and is a major health concern in the Philippines. To determine the dengue disease burden in the Philippines and identify gaps and future research needs, we conducted a literature analysis and review to describe the epidemiology of dengue disease. We used well-defined methods to search and identify relevant research conducted between 2000 and 2011. This long-term review highlights an increase in the reported incidence of dengue disease in the Philippines. The rising incidence of dengue disease may be related to a growing population, increasing urbanization, improvements in surveillance, and the limited success of vector control measures. Gaps in the epidemiological information available in the Philippines during the period 2000–2011 include comprehensive national and regional data that describe the proportion of severe dengue disease, including hospitalizations and mortality, and incidence data per 100,000 population. More comprehensive data are also needed for age, serotype, and seroprevalence on both national and regional levels. The data presented enable the observation of epidemiological characteristics, both within and across years. Such assessments are essential at national and regional levels to improve both preparedness and response activities relating to dengue disease outbreaks.

## Introduction

Dengue is a growing health concern in the Philippines. Outbreaks were reported in1926 [1], [2], and the first recorded epidemic in Southeast Asia occurred in Manila in 1954 [3], [4]. Further epidemics occurred in 1966, 1983, and 1998, with increasing reported cases of dengue disease [5]–[8]. The 1998 epidemic had the highest recorded incidence rate (60.9 cases per 100,000 population) and case fatality rate (CFR; 2.6%) [5]. The rising incidence of dengue disease can be explained by several factors. Dengue is caused by one of four dengue viruses (DENV-1, -2, -3, or -4) transmitted primarily by the *Aedes aegypti* (Linnaeus) mosquito, which breeds in open water containers, and can survive year round in tropical and subtropical climates. During World War II, the movement of people and equipment expanded the geographic distribution of *Ae. Aegypti* and dengue disease in Southeast Asia [3]. Since then, virus propagation in the region has been facilitated by rapid urbanization, environmental degradation, the lack of a reliable water supply, and improper management and disposal of solid waste [3], [9]. In the Philippines, the percentage of the population living in urban areas increased from 27.1% in 1950 to 58.5% in 2000 [10].

Dengue has been a notifiable disease in the Philippines since 1958 [11]. During the review period the Philippines employed both passive (outpatient and inpatient) and sentinel surveillance across all ages [12]. Prior to 2006, the National Epidemic Sentinel Surveillance System, managed by the National Epidemiology Center (NEC) of the Department of Health (DoH), maintained surveillance of notifiable diseases, including dengue disease. The National Epidemic Sentinel Surveillance System monitored the total number of hospital cases and deaths that were admitted to 250–400 selected sentinel hospitals throughout the Philippines and, up until 2005, did not differentiate between dengue fever (DF), dengue haemorrhagic fever (DHF), or dengue shock syndrome (DSS). To improve surveillance, in 2005, the system changed to separate reporting of DF, DHF, and DSS. In 2007, the Sentinel Surveillance System was expanded to include up to 1662 disease reporting units (including sentinel hospitals, private hospitals, and rural health facilities) to develop an all-case (suspected and probable) reporting system (Philippines Integrated Disease Surveillance and Response System). In addition, virological surveillance of dengue disease was implemented in 2008 [13]. The Field Health Surveillance Information System, also managed by the NEC of the DoH, is a passive reporting system that consolidates public health statistics due to notifiable diseases, including dengue disease, from all levels of government health facilities in the Philippines.

Most reported dengue cases are suspected or probable cases according to standard definitions and are not laboratory confirmed. In a recent review of research needs for dengue surveillance and emergency response [14] (an update of a review of surveillance systems in dengue-endemic countries by Gubler [15]) laboratory capability for DENV serology was rated as ‘good’ in the Philippines but laboratory capability for dengue disease virology was rated as ‘exists’ (rather than ‘good’) [14]. In addition, at the time of the review there was a lack of laboratory capacity to confirm cases [16]. The 2009 World Health Organization (WHO) classification of dengue by levels of severity is currently used in the Philippines: non-severe dengue with or without warning signs, and severe dengue (severe plasma leakage, severe bleeding, or severe organ involvement) [17]. An objective of the Philippines DoH is to ensure that this system is consistently applied in the country. However, at the end of 2012, the 1997 WHO classification [18] continued to be used by some reporting physicians. This older system grouped symptomatic dengue into three categories: undifferentiated fever, DF, and DHF; DHF was further classified into four severity grades, with grades III and IV being defined as DSS [18].

**Figure 1:**
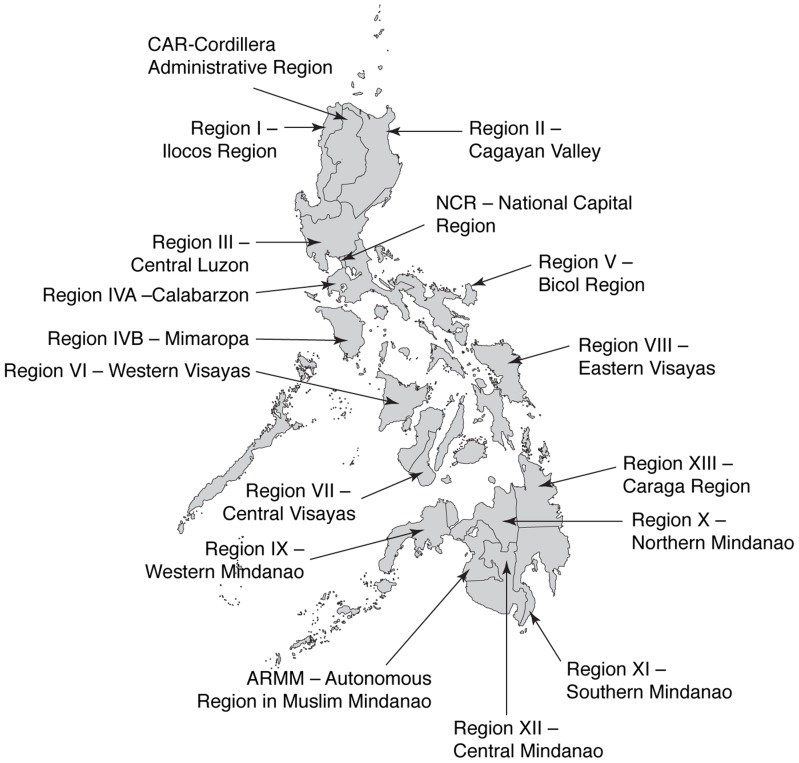
Map of the Philippines showing the administrative 17 regions [19], [20]. The Republic of the Philippines is an archipelago in Southeast Asia consisting of 7107 islands. The country is divided into 17 regions within the three island groups of Luzon (Regions I–V, Cordillera Administrative Region [CAR] and National Capital Region [NCR]), Visayas (Regions VI–VIII) and Mindanao (Regions IX–XIII and Autonomous Region in Muslim Mindanao). Metro Manila is the metropolitan area that contains the City of Manila, the capital of the Philippines. The metropolis is officially called the National Capital Region (NCR, the term used throughout this report) and is composed of Manila plus 16 neighboring cities and municipalities, including Quezon City.

The Republic of the Philippines comprises 7107 islands in Southeast Asia; the three island groups of Luzon (Regions I–V, Cordillera Administrative Region [CAR], and National Capital Region [NCR]), Visayas (Regions VI–VIII), and Mindanao (Regions IX–XIII and Autonomous Region in Muslim Mindanao) are split into 17 regions (**Figure 1**). The population is 92,337,852 (2010 census), with an average annual population growth rate of 1.90% for the period 2000–2010 [19]. A large proportion of the population (37.3%) lives in three regions: Calabarzon (Region IV-A; 11.74 million people), the capital, Metro (metropolitan) Manila, also known as NCR (11.55 million people), and Central Luzon (Region III; 9.72 million people) [20]. The Philippines has a tropical marine climate, with an average annual temperature of 27°C, annual dry seasons from December to May, and annual wet seasons from June to December [21].

A systematic literature review was conducted to describe the available epidemiology of dengue reported in the Philippines between 1 January 2000 and 23 February 2012. Our objectives were to describe the recent epidemiology of dengue (national and regional incidence [by age and sex], seroprevalence and serotype distribution and other relevant epidemiological data) and to identify gaps in epidemiological knowledge requiring further research, but not to provide an exhaustive picture of the history of dengue in the country. Given the 3–5-year periodicity of dengue outbreaks [3] we estimated that at least one decade of data would be necessary to provide an accurate image of recent evolution of epidemiology. Furthermore, a 10 year period was determined to observe serotype distribution over time and through several epidemics. For convenience, we chose to start our review period on 1 January 2000 and set the cutoff as 23 February 2012, the date when we initiated this review. An additional rationale for selecting 1 January 2000 as the start date for this review, as opposed to an earlier date, was that we hypothesized that this would limit the bias that any differences in surveillance practices over time would have on the results.

## Materials and Methods

A Literature Review Group developed a protocol for this literature survey and analysis based on the preferred reporting items of systematic reviews and meta-analyses (PRISMA) guidelines [22]. The protocol was registered on PROSPERO, an international database of prospectively registered systematic reviews in health and social care managed by the Centre for Reviews and Dissemination, University of York (18 May 2012; http://www.crd.york.ac.uk/PROSPERO/display_record.asp?ID=CRD42012002292).We utilised an inclusive search strategy to find papers, theses, dissertations, reports and statistical tables, as well as official web sites and grey materials. The Literature Review Group defined the inclusion/exclusion criteria and guided the search and selection process described below. Decisions were made by reaching a consensus via teleconferences. It was expected that the resulting articles would be heterogeneous with respect to data selection, and classification of cases, and would not be methodologically comparable. We therefore planned not to perform a meta-analysis.

### Search strategy and selection criteria

Search strings for each database were designed with reference to the expanded Medical Subject Headings thesaurus, encompassing the terms ‘dengue’, ‘epidemiology’, and ‘Philippines’. Different search string combinations were used for each electronic database with the aim of increasing the query's sensitivity and specificity.

Only studies published in English between 1 January 2000 and 23 February 2012 were included. For databases that did not allow language and/or date limitations, references not meeting these criteria were deleted manually at the first review stage. No limits by sex, age and ethnicity of study participants or by study type were imposed, although single-case reports were excluded, as were studies that only reported data for the period before 1 January 2000. As duplicate publication of data (e.g., in meta-analyses and other reviews) could lead to oversampling and overestimates of the incidence of dengue disease, literature reviews and editorials involving previously published peer-reviewed data were also excluded.

In March 2012 we searched the following databases (1) PubMed (http://www.ncbi.nlm.nih.gov/pubmed/); (2) WHO Library database (WHOLIS: http://www.who.int/publications/en/); (3) WHO Western Pacific Region (WPRO: http://www.wpro.who.int/en); (4) Index Medicus for South-East Asia Region (IMSEAR: http://imsear.hellis.org/); (5) WHO Regional Office for Southeast Asia (WHOSEAR: http://www.searo.who.int/en/); (6) Philippines Ministry of Health official bulletins (http://www.doh.gov.ph/); (7) Philippines National Institute of Health (http://nih.upm.edu.ph/); (8) Philippine Council for Health Research and Development (http://www.pchrd.dost.gov.ph/); and (9) National Epidemiology Center, DoH, Philippines.

We also included data from several other sources to complement articles identified by the primary literature review: two national journals (the Pediatric Infectious Disease Society of the Philippines journal; http://pidsphil.org, and the Philippine Society for Microbiology and Infectious Disease journal; http://www.psmid.org.ph/) and The Western Pacific Surveillance and Response (WPSAR; http://www.wpro.who.int/wpsar/en/) open-access journal dedicated to the surveillance of and response to public health events were searched; other reports and guidelines published on-line by relevant organizations; conference papers and posters from infectious disease, tropical medicine, and paediatric conferences, and grey literature (e.g., lay publications) were sought through general internet searches (e.g., Google and Yahoo; limited to the first 50 search results). Additional publications and unpublished data sources meeting the search inclusion criteria were included if recommended by a consensus of the Literature Review Group.

After removing duplicate citations, the Literature Review Group reviewed the titles and abstracts and identified those for which the full text was retrieved. A second review was performed on the full text to make the final selection of relevant articles to include. Studies were reviewed by the Literature Review Group to ensure they complied with the search inclusion and exclusion criteria. In particular, publications of duplicate data sets were excluded, unless the articles were reporting different outcome measures. We chose not to exclude articles and other data sources nor formally rank them on the basis of the quality of evidence. Indeed while it is recognized that assessing study quality can potentially add value to a literature review, the consensus of the Literature Review Group was that given the expected high proportion of surveillance data among the available data sources and the nature of surveillance data (passive reporting of clinically-suspected dengue), such quality assessment would not add value in this case. As our primary objective was to describe the recent evolution of dengue, rather than to quantify disease in absolute terms, we therefore retained all available data sources.

The selected data sources were collated and summarized using a data extraction instrument developed as a series of Excel (Microsoft Corp., Redmond, WA) spreadsheets. Data were extracted into the spreadsheets according to the following categories for analysis: incidence, age, sex and serotype distribution, serotype data, seroepidemiology or seasonality and environmental factors, by national or regional groups. Data from literature reviews of previously published peer-reviewed studies and pre-2000 data published within the search period were not extracted. The original data sources and the extraction tables were made available to all members of the Literature Review Group for review and analysis.

### Amendment to protocol

Additional data on dengue were provided by the Philippines DoH NEC on 28 May 2012 [23]. The NEC Library, formerly the Field Epidemiology Training Program (FETP) Library (http://nec.doh.gov.ph/index.php?option=com_content&view=article&id=43&Itemid=58), established in 1989 under the FETP project of the DoH, contains reports of local infectious disease outbreaks submitted to them by FETP Fellows and by Regional Surveillance Units. The reports of dengue disease outbreaks that occurred between 1987 and 2011 were manually searched, and data were collated and summarized using the data extraction instrument. The NEC also provided data from the urban area of Quezon City, NCR, and the rural area of Rizal, Region IV-A. Data included the population numbers and the number of dengue disease cases by year (1999–2011) and by age (<1 year, 1–10 years, 11–20 years, 21–30 years, 31–40 years, >40 years). These data were integrated with the other data sources in the data extraction tool.

## Results

**Figure 2:**
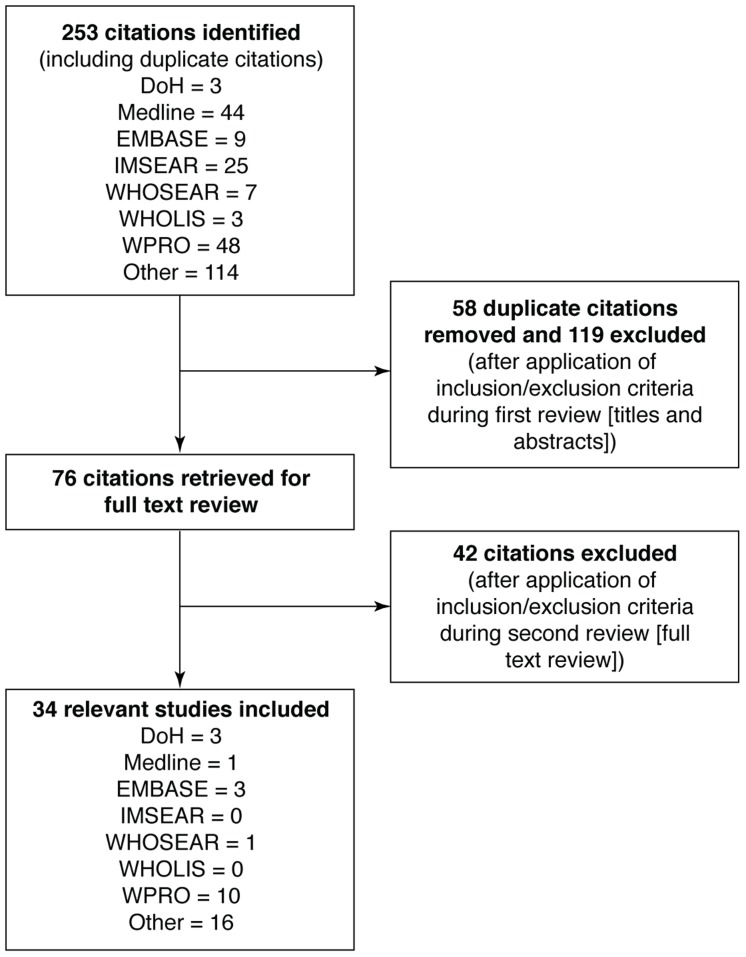
Results of literature search and evaluation of identified studies according to PRISMA. All references identified in the on-line database searches were assigned a unique identification number. Following the removal of duplicates and articles that did not satisfy the inclusion criteria from review of the titles and abstracts, the full papers of the first selection of references were retrieved either electronically or in paper form. A further selection was made based on review of the full text of the articles. DoH, Department of Health; EMBASE, Excerpta Medica Database; IMSEAR, Index Medicus for South East-Asia Region; Medline, United States National Library of Medicine and the National Institutes of Health Medical Database; PRISMA, preferred reporting items of systematic reviews and meta-analyses; WHOLIS, World Health Organization Library database; WHOSEAR, World Health Organization Regional Office for Southeast Asia; WPRO, World Health Organization Western Pacific Region.

The searches identified 253 citations, and of these, 34 sources fulfilled the inclusion criteria (**Figure 2** **; Table S1**). Of the 17 articles, there were 14 publications in peer reviewed journals, and these described data from seven geographical locations, mostly in or around NCR. Three posters presented data from Cebu, Western Visayas but these data had not been formally published at the time of this review (note these data have recently been published in the 2012 Dengue bulletin [24]). The study design for these articles varied, but the majority were prospective (n = 5) or retrospective (n = 4) surveillance studies (**Table S1**). As can be seen from Table S1, the remaining data sources were either DoH or WHO reports that were found during the initial searches or statistical tables recommended or accessed by members of the LRG to supplement incomplete data presented in the reports. A narrative synthesis of our findings is presented.

### National epidemiology

**Figure 3:**
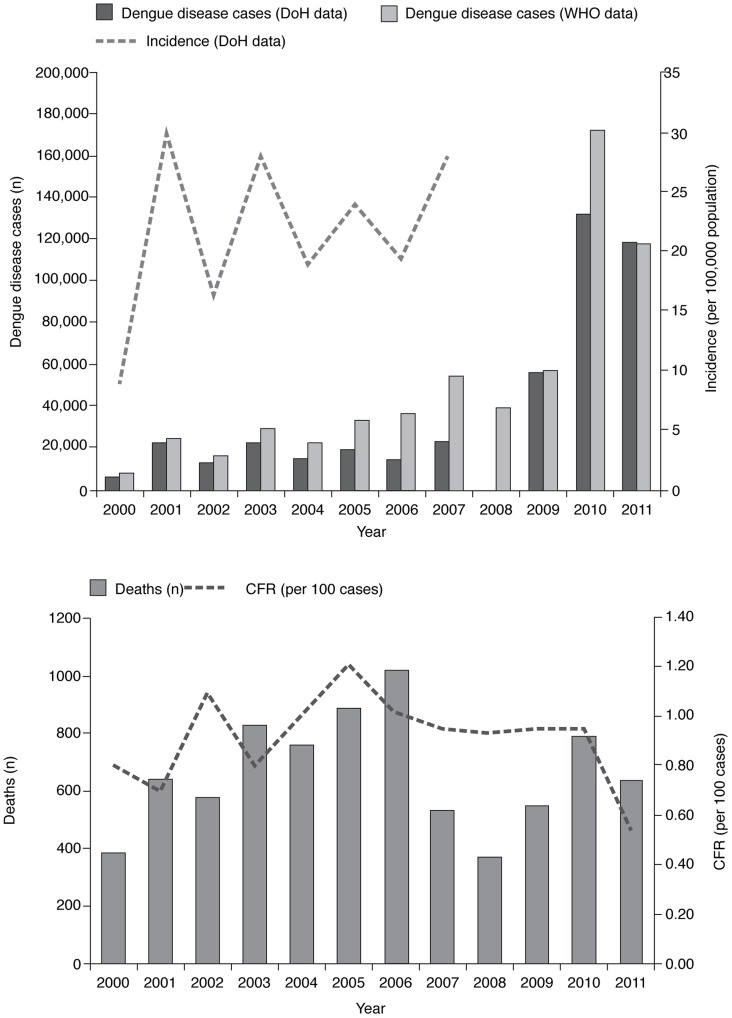
The epidemiology of dengue disease in the Philippines, 2000–2011. A: The number of reported dengue disease cases and incidence per 100,000 population. B: The number of reported deaths attributed to dengue disease and CFR per 100 cases. The reported number of dengue disease cases in the Philippines fluctuated throughout the review period, with an overall increase in incidence observed over time. Peaks in dengue disease cases occurred in 2001, 2003 and 2007. Dengue disease-related deaths fluctuated, peaking in 2006. Overall, the CFR was in the range 0.5–1.2 per 100 cases and decreased after 2005. Using available data* from the DoH [5], [25]–[29] and the WHO [6], [16], [20], [21], [30]–[32]. Sources: Number of reported cases: 2000–2005: DoH 2000–2005 [25]; 2006–2007: FHSIS 2000–2009 [29]; 2008–2011: WHO data: WHO 2008 [16], 2009 [20], 2012 [31], [32] (2010 and 2011 values estimated from graph); Incidence: 2000–2007: FHSIS 2000–2009 [29]; Deaths: 2000–2005: DoH 2000–2005 [25]; 2006: DoH 2011 [28]; 2007–2010: Arima and Matsuia 2011 [33]; 2011: WHO 2012 [31] (value estimated from graph); CFR: 2000–2005: WHO 2008 [16]; 2006–2010: Arima and Matsuia 2011 [33]; 2011: WHO 2012 [31] (value estimated from graph). *Data on all dengue disease cases were not publically available from the DoH between 2008 and 2011. CFR, case fatality rate; DoH, Department of Health; WHO, World Health Organization.

Among the included sources, no complete and comparable data were found for the entire review period. The most complete datasets for the number of cases of dengue disease in the Philippines and the number of dengue-related deaths were reported by the DoH [5], [25]–[29] and the WHO [6], [16], [20], [21], [30]–[32] (**Figures 3A** **and** **2B** **, Table S2 and Table S3**), although a number of sources did report similar, but isolated, data during the survey period [13], [33], [34]. An overall summary plot of these data would be of little value in identifying trends over time. Despite possible bias therefore it is useful to view the data made available during the review period from the WHO and DoH (**Figures 3A and 3B**). These data show that the reported number of dengue disease cases fluctuated throughout the review period, with an overall increase in cases observed over time (**Figure 3A**). There was a sharp rise in the number of cases in 2001 (23,235 cases) compared with the previous and following year, and in a similar fashion high numbers of cases were also reported in 2003 (22,789 cases) and 2007 (23,773 cases) as shown by DoH data. The incidence per 100,000 population was 30 cases in 2001, 28.1 cases in 2003, and 28.2 cases in 2007 [5], [25], [29]. Possibly as a result of data extrapolation from incomplete submissions from some regions, the WHO data showed consistently higher numbers of cases than the DoH, but the same general pattern. A large increase in the number of cases was recorded in 2010, with 131,976 and 173,033 cases reported by the DoH and the WHO, respectively, compared with 56,545 and 57,819 cases, respectively, in 2009 [30]–[33]. There were also a large number of cases reported by the DoH in 2011 (118,868) [31]. Overall, the CFR ranged from 0.5% to 1.7% [5], [6], [16], [29], [30], [32]. There were 548 fatal cases in 2009 (CFR 0.95%), increasing to 788–793 in 2010 (CFR 0.60–0.94%) [30], [32], [33].

Data on the severity of dengue disease cases were inconsistently reported over the review period. However, the available data from the DoH showed an increase in the number of DHF/DSS cases reported and the incidence of DHF/DSS per 100,000 population in the middle of the decade (2006–2008: 11,915–14,310 DHF/DSS cases, 14.1–17.7 per 100,000 population) [29]. Data from the DoH also showed that peaks in the number of dengue disease-related deaths were observed at the beginning of the decade (2001: 641 deaths) and from 2003 to 2006 (2003: 831 deaths; 2004: 761 deaths; 2005: 887 deaths; 2006: 1017 deaths). Overall, the CFR was in the range 0.5–1.7% (DoH) or 0.5–1.2% (WHO) and decreased after 2005 (**Figure 3B**).

### Regional epidemiology

The numbers of dengue disease cases being reported were highest in the most populated urban areas, such as NCR [25], [28], [29], [35]–[40]. However, the incidence of dengue disease per 100,000 population varied by year and by region. The dengue disease incidence rates per 100,000 population were highest in the NCR in 2000, CAR in 2001, Region VI in 2002, Region VII in 2007, Region XI in 2003, 2004, 2008 and 2009, and Region XII in 2005 and 2006 (**Table S4**) [25], [29]. Incidence rates for 2010 and 2011 were not available. However, in 2010, the highest number of cases by region was in Western Visayas (Region VI; 17,593 cases; 84 deaths; CFR 0.48%) [36]. In 2011, the highest number of cases by region was in NCR (15,427 cases; 93 deaths; CFR 0.60%), and the NCR area with the highest number of cases was Quezon City (4611 cases; 32 deaths; CFR 0.69%) [36]. The highest numbers of fatal dengue disease cases were in the NCR in 2001, 2003, 2004, 2005, and 2006 (121, 148, 131, 185, and 345 cases, respectively) [28]. By contrast, in 2002, the region with the highest number of fatal dengue disease cases was Central Visayas (Region VII; 107 cases) [28].

The numbers of reported dengue disease cases were substantially higher in Quezon City than in Rizal, even though the populations are similar: 2.7 million and 2.5 million in Quezon City and Rizal, respectively, in 2011 [23]. Nevertheless similar patterns of reported dengue were seen in both regions with an increase in cases over the study period and higher numbers of cases in 2006, 2008, 2010 and 2011 compared with other years. The DoH has also recorded local outbreaks of dengue disease from 2000 from FETP Fellows reports and from Regional Surveillance Units reports [23]. At least one local outbreak was reported in each year and in all regions except Regions VIII and XIII. The regions with the highest numbers of local outbreaks reported were CAR (10 outbreaks), Region III (five outbreaks), Region IV (four outbreaks), and Region X (four outbreaks). The outbreak with the highest number of dengue disease cases was reported in Zamboanga City, Region IX, in 2010 (2122 cases; 22 fatal cases; CFR 1.04%).

### Demographic patterns

Where data were available over the review period, children aged 5–14 years old represent the age group with the highest proportion of dengue disease in the Philippines (**Table S5**) [6], [20], [25], [29], [36], [41], [42]. Dengue disease cases were reported by age group to the DoH in 2000–2003 and in 2005–2009. In 2000–2003 and 2005–2009, the highest proportions of cases were reported in individuals who were 5–14 years old (28.6–50.6% of cases), followed by those who were 15–49 years (21.2–37.3% % of cases) and 1–4 years (15.4–31.1% of cases) [29]. In 2010–2011, the largest proportion of dengue disease cases reported to the DoH was in individuals aged 1–10 years (around 25,800 of 70,204 [36.8%] cases (value estimated from Figure 2 in Disease Surveillance Report, DoH, 2011 [36]).

Where incidence data were available, the highest rates in 2000, 2003, and 2005 were reported in individuals who were 5–14 years old, followed by those who were <5 years old, and then 15–49 years old. In 2006, the highest incidence rates were reported in individuals who were 0–4 years old, followed by those who were 5–14 years old, and then in those who were 15–49 years old (**Table S5**).

In both Quezon City (NCR) and Rizal (Region IV-A), there was a general increase in the numbers of dengue disease cases reported over time in each age group. The highest numbers of cases were reported in individuals aged 1–9 years, followed by 10–19 years, with these two age groups representing over 75% of reported cases in each year [23]. Furthermore, only 1% of patients with dengue admitted to San Lazaro Hospital, in Manila, Luzon were over the age of 35 years [43]. The WHO reported similar findings with respect to dengue disease incidence by age: in 2008, of 7880 patients with dengue disease admitted to different sentinel hospitals nationwide from 1 January to 29 March, the median age was 12 years (range <1 month to 87 years) [20].

A prospective community-based study of dengue disease in infants 2–15 months of age was conducted from January 2007 to May 2009 in the semi-urban community of San Pablo, Laguna, Calabarzon (Region IV-A). Between January 2007 and January 2008, the modal age for symptomatic dengue disease in these infants was 8 months (median 7.2 months) [44]. The age-specific incidence of infant DHF was 0.5 per 1000 persons aged 3–8 months and zero among those aged ≥9 months [44].

The DoH has reported the numbers of dengue disease-related deaths by age group in 2003–2005 [25]. Over 80% of the fatal cases in each year occurred among individuals aged <20 years [25]. Among those aged <10 years, there were 477–562 fatal cases in 2003–2005 (62.7–66.1% of all dengue disease-related deaths). Data from the WHO showed that the majority of dengue disease-related deaths occurred among children aged <9 years [6].

Few data were available for CFR by age. Available data for 2003 and 2005 showed that the CFR decreased from age 0–4 years (CFR range 0.29–0.37%) to 5–14 years (0.18–0.23%) and to 15–49 years (0.09–0.13%). By contrast, CFR increased for the age groups 50–64 years (0.13–0.17%) and ≥65 years (0.42–0.95%).

Data regarding the sex distribution of dengue disease in the Philippines are scarce and thus it is difficult to discern any distribution pattern. In one report from the WHO (covering the period 1 January to 29 March, 2008), the majority of dengue cases (53%) were male [20]. Similar proportions were found in the analysis of urban Quezon City versus rural Rizal in 2000 to 2011 [23]. Another WHO report suggested that dengue disease cases and related deaths occur in approximately equal proportions among males and females [16]. The only other data found were from a 10-month, prospective cohort study of 42 dengue disease patients admitted to a tertiary referral hospital in Cardinal Santos Medical Center, San Juan (NCR), from November 2006 to August 2007, in which dengue disease occurred in more females (57.1%) than males [45].

### Seroepidemiology

Few studies show any analysis of the seroepidemiology of dengue. Nevertheless, a longitudinal prospective cohort study of fever in 4441 infants in San Pablo Laguna conducted from January 2007 to May 2009, showed that 11% of all presenting undifferentiated febrile illness was dengue (40 cases of dengue diagnosed out of 353 cases of fever). All cases of dengue but one were primary dengue (as determined using IgG/IgM and paired sera) and DHF was seen only in infants under 8 months of age. DENV-3 predominated in this cohort (as noted below) and infants with high levels of anti-dengue 3 antibodies at birth developed dengue infections later than infants with low levels of antibody at birth. The overall infection rate (as determined by seroconversion in a subset of this cohort) was nearly 12%, between January 2007 and January 2008, and 87% of these dengue infections were asymptomatic or only mildly symptomatic [44], [46] (**Table S1**). In a prospective study of children admitted with fever without a clear focus to St Lukes Medical Centre in Quezon City, Metro Manila, from January 1999 to December 2001, 71.4% had dengue (confirmed by IgM and/or RT-PCR) and 1/3 had DHF [47], [48]. Furthermore, in another prospective fever surveillance study of patients with a mean age of 18 years admitted to San Lazaro Hospital, in Manila, Luzon, 87% of those with fever without a clear focus of infection had dengue, 7% of the cases were primary dengue infections (determined by IgM/IgG ELISA) [43].

### DENV serotype distribution

**Figure 4:**
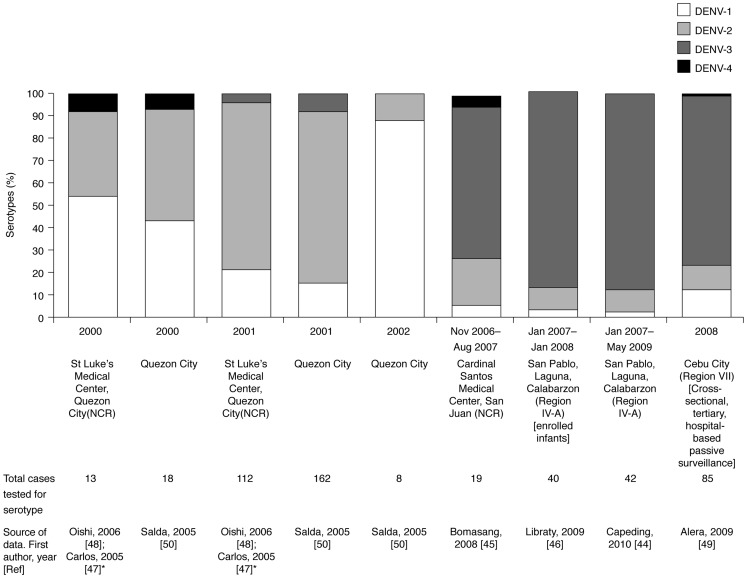
Dengue virus serotype distribution in the Philippines: regional studies. All four DENV serotypes were reportedly present in the Philippines at some time during the review period [43]–[50] but the predominant serotypes changed from DENV-1 and -2 early in the review period [47], [48], [50] to DENV-3 towards the end of the review period [43]–[46], [49].

Studies published during the review period that observed DENV serotypes were hospital or community based and involved low numbers of cases; no published studies examined national or regional serotype distribution. All four DENV serotypes were reportedly present in the Philippines at some time during the review period (**Figure 4**) [43]–[50]. DENV-1 and -2 appeared to be more prevalent (2000–2001) in a prospective study of hospitalized paediatric patients in NCR (January 1999 to December 2001) [47], [48] and in isolates from dengue disease outbreaks in the Philippines (1995–2002) [50]. In studies towards the end of the review period DENV-3 became more predominant [43]–[46], [49], DENV-4 was either not present [44], [46]–[48], [50], or was present in up to 7% of the dengue disease cases [45], [49], [50] in the studies included in this review.

The molecular epidemiology of DENV-2 isolated from patients with DF, DHF and DSS in the Philippines between 1995 and 2002 was examined by Salda et al. who have shown evolution from the Asian 2 genotype to the Cosmopolitan genotype, first identified in their sample of virus isolates in 1998 [50]. The genetic sequence of DENV-3 circulating in 2008–2010 outbreaks was characterized by Destura et al. to help assess the relationship between genotype mutations and the potential to cause outbreaks of severe or attenuated disease. The isolates did not fall into any of the groups of the reported genotypes suggesting the identification of a new genotype [51].

### Seasonality and environmental factors

The LRG were aware of data that suggests the number of cases increasing 1–2 months after the onset of the rainy season, resulting in a peak of dengue cases in July to November, especially August. However, these are unpublished statistics and thus there is a gap in the available data that can address the influence of seasonal factors on the incidence of dengue disease. One study that assessed climactic factors associated with dengue disease incidence showed that high rainfall (but not temperature) was significantly associated with increased dengue disease incidence in Metro Manila in 1996–2005 [40]. Local outbreak data from FETP Fellows reports and from Regional Surveillance Units reports showed that dengue disease incidence was linked to flooding and/or to the unsuitable storage of water, e.g., in open containers, which are potential breeding areas for mosquitoes [23].

## Discussion

This systematic review provides a comprehensive overview of the available data relating to the epidemiology of dengue in the Philippines for the period 2000–2011, and highlights many areas for further study. The national surveillance system has faced challenges over the period of the review related to the reliability and consistency of data collection and reporting. Furthermore, relative to other countries in the region, for example Thailand and Vietnam, the amount of published dengue epidemiological research is limited. Nevertheless, this review did reveal some interesting data.

### Seasonal and environmental factors

Seasonal and environmental factors affecting dengue in the Philippines are under-studied. Only one study attempts to correlate climatic factors with annual peaks in dengue cases, and this study was based in metro Manila. The Philippines has four distinct climate types across its extensive and diverse geography and further study is needed to better understand the seasonal patterns affecting the whole country.

### Demographic findings

The high proportion of dengue disease and related deaths reported in children versus other age groups may reflect the age profile of the population; approximately one-third of the population is aged <15 years. Additionally, because dengue is highly endemic in the Philippines, most adults are immune. Understanding age distribution of dengue disease can aid the identification of groups with a high risk of dengue disease, provide information on age-related severity. From the data used in this review, no conclusions can be drawn regarding the sex distribution of dengue disease in the Philippines.

### Severity of dengue disease

Comprehensive national and regional data that describe the proportion of severe dengue disease cases, including hospitalizations and mortality, are lacking. The incidence of the severe forms of the disease, DHF/DSS, appeared to increase in the middle of the decade. The number of dengue disease-related deaths varied throughout the Philippines, with several peaks observed. Interestingly, the CFR also fluctuated. There are several potential reasons for this observation, including greater public awareness and early case detection, the possibility that certain dengue serotypes/genotypes cause less severe disease, and variations in clinical case management of dengue disease during 2000–2011. Another important reason why severe cases may have spiked midway through the decade is that prior to 2005 the reporting forms did not facilitate the reporting of DHF and DSS separately from DF. The introduction of the 2009 WHO classification of dengue disease [17] may improve the completeness of severity data in the Philippines although it may take, several years before the majority of both public and private institutions use this classification and report their data accordingly.

### DENV serotype distribution

All four serotypes were present during 2000–2011, with the co-circulating types varying temporally and spatially. However, few data were available, and study findings do not represent the national or regional distribution. The data suggest a shift towards a prevalence of the DENV-3 serotype towards the end of the review period. However, studies assessing DENV serotype distribution were mainly hospital or community based and involved low numbers of cases; no published studies examined national or regional serotype distribution. Robust surveillance of serotype distribution is essential to monitor changes in the relative prevalence of DENV serotypes (or their variants) and any potential effects this may have on dengue disease incidence or severity and to help predict epidemics.

### Seroepidemiology

The presenting signs and symptoms in individuals with dengue disease are similar to those with other non-dengue acute undifferentiated febrile illnesses. Thus, in dengue-endemic regions clinicians should maintain a high index of suspicion for dengue disease. Available data on the laboratory confirmation of dengue disease cases were scarce, but showed that DENV antibody tests were used for most confirmed cases. Additionally, the few prospective fever surveillance studies that were reported during the review period showed that a variable proportion of patients with fever presenting to, or admitted to, hospital had dengue. This variation was most likely related to differences in study design, and a high proportion of children and adults admitted to hospital with fever, without a clear focus of infection had dengue. Serological analysis showed that most children and adults admitted to hospital were experiencing a secondary dengue infection (determined by IgM/IgG ELISA). The only study to determine an incidence of infection (in infants), showed an incidence of 12%, which is comparable to that seen in other prospective studies in highly endemic countries [52]. In the study by Capeding et al., all cases of DHF were observed in younger infants (aged <8 months) [44], consistent with previous observations that younger age groups are particularly vulnerable to severe disease [53].

### Evidence gaps

Understanding the spectrum of dengue disease is essential to combating the disease. However, gaps in the epidemiological information available during 2000–2011 have been highlighted in this review and provide indications for avenues of future research. Comprehensive and continuous data are lacking for the review period, in particular national and regional age-stratified incidence rates and sex distribution data. This limits the possibility of making comparisons and drawing firm conclusions over the years, across regions, and among different ages. Although data are available on the number of dengue disease cases nationally and regionally, there are relatively few reported data on the incidence of dengue disease per 100,000 population. There were relatively few published studies of regional data on dengue disease epidemiology for the period 2007–2009. Availability of comprehensive data sets would allow a more systematic evaluation of the trends and informed assessments about their impact on surveillance procedures and control measures.

Further studies exploring DENV serotype distribution and seroprevalence data as well as the associations between DENV serotype and disease severity are necessary. For example, studies in some countries have demonstrated that DENV-3 is associated with a significant proportion of severe complications, and the displacement in the predominant serotype has been related to local outbreaks of disease [54]. This knowledge can help guide the introduction of additional public health measures, including vector control intervention, educational communication, and the adequate provision of medical supplies. Estimates of the extent of dengue disease under-reporting would also be valuable.

Whilst the epidemiology of dengue disease varied between the three island groups both spatially and temporally, the data do not reveal any geographical patterns at an island level in incidence or other epidemiologic parameters. Although the regions with the highest incidence, morbidity, and mortality were generally urban centres, regions with the highest incidence rates and peaks in the number of dengue disease-related deaths and the intensity and magnitude of dengue cases changed each year. The Philippines is severely affected by extreme weather events and is vulnerable to climate change. Vector-borne diseases, such as dengue disease, may be particularly sensitive to both periodic fluctuations and sustained changes in global and local climates. A programme of regional dengue disease burden surveillance studies will provide scientific data on which to base decisions regarding priorities, resource allocation, and geographical areas for targeting vector control. In addition, studies on the effects of continuing urbanization (including the effects of human density and population movement) as well as information relating to the effects of housing conditions, water supplies, and waste management on the incidence of the disease would be useful.

### Limitations and strengths of the review

Strengths of this systematic review include the complementary information provided from national surveillance data and local studies. However, there are several limitations to note. There is a general scarcity of published information on the epidemiology of dengue disease in the Philippines. Additionally, some of the studies identified may have weaknesses, such as inadequately described case selection and a lack of sound statistical methods, which were not accounted for as no assessment of quality of evidence was conducted. Another limitation of the data generated by this review is the discrepancies in the reported dengue disease rates between the WHO and the DoH. As already noted, this may be due to data extrapolation from incomplete submissions from some regions of the Philippines. There are also inherent limitations associated with the surveillance data due to changes in reporting behaviour, the systems used, misclassifications, and under-reporting [3], [13]. Importantly, a proportion of the increase in the number of dengue disease cases towards the end of the review period may be an artefact of the changes in the surveillance system, including the separate reporting of DF, DHF, and DSS since 2005, and the transition to the all-case reporting surveillance system with the increase from 250–400 sentinel hospitals to a network of up to 1662 disease reporting units since 2006 [33]. Another limitation of this review is that the number of cases of dengue disease may be under-reported by the surveillance system in the Philippines. Although some hospitals may over-diagnose dengue disease cases, there may be an overall under-reporting of cases due to variability in defining dengue disease, the passive surveillance system used in the Philippines [3], [13], the sentinel system used prior to 2006 [33], and the exclusion of data from privately treated patients. Different applications or interpretations of case definitions over the review period limit the ability to make valid temporal comparisons.

### Conclusions

This long-term review highlights an increase in the reported incidence of dengue disease in the Philippines. All regions reported cases of dengue disease, although more cases were reported from the most populated, urbanized areas. The reported number of cases of dengue disease fluctuated throughout 2000–2011, with an overall increase in cases over time. The highest incidence of dengue disease cases per 100,000 population was reported in children 5–14 years of age, followed by children 0–4 years old, and 80% of all dengue disease-related deaths were reported in individuals aged <20 years. In the regions of the Philippines, the incidence of dengue disease per 100,000 population varied, with particularly high incidences observed in the regions of the island of Mindanao. The increasing incidence of dengue disease may be related to a growing population, increasing urbanization, improvements in surveillance, and the limited success of vector control measures.

All four DENV serotypes were present; however, there was a shift to DENV-3 towards the end of the literature review period. Recent improvements to the surveillance system and more consistent use of the 2009 WHO classification of dengue disease [17] may help standardize the approach to data collection and reporting of dengue disease in the Philippines.

## Supporting Information

Table S1 **Evidence table for citations fulfilling the inclusion and exclusion criteria for the literature review (n = 34).**
(PDF)

Table S2 **Number and incidence of dengue disease cases in the Philippines: national data.** DF, dengue fever; DHF, dengue haemorrhagic fever; DoH, Department of Health; DSS, dengue shock syndrome; FHSIS, Field Health Surveillance Information System; NSCB, National Statistical Coordination Board; WHO, World Health Organization. *Values estimated from graphs.
(PDF)

Table S3 **Dengue disease-related deaths and case fatality rate in the Philippines: national data.** CFR, case fatality rate; DF, dengue fever; DHF, dengue haemorrhagic fever; DoH, Department of Health; DSS, dengue shock syndrome; WHO, World Health Organization. *Values estimated from graphs. ^†^Cases in children (5–9 years old only). ^‡^Cases in children (10–14 years old only).
(PDF)

Table S4 **Number and incidence of dengue disease in the Philippines.** (A) Regional data; (B) outbreak data from Field Epidemiology Training Program Fellows' reports; (C) outbreak data from Regional Surveillance Units reports. ARMM, Autonomous Region in Muslim Mindanao; CAR, Cordillera Administrative Region; CFR, case fatality rate; DF, dengue fever; DoH, Department of Health; FHSIS, Field Health Service Information System; NCR, National Capital Region; NDRRMC, National Disaster Risk Reduction and Management Council. *1 January to 10 September, 2010 or 2011. ^†^January to August, 2011. ^‡^Ongoing outbreak.
(PDF)

Table S5 **Age-specific patterns of dengue disease in the Philippines** **[25], [29]** **.** Population data from: http://www.doh.gov.ph/kp/statistics/demography1.html. Empty cells: data not provided. DF, dengue fever; DHF, dengue haemorrhagic fever; DoH, Department of Health; FHSIS, Field Health Service Information System; NSCB, National Statistical Coordination Board. *http://www.nscb.gov.ph/secstat/d_popnProj.asp.
(PDF)

Checklist S1 PRISMA 2009 checklist.
(PDF)
