# Supplementary material for: Epidemiology of Dengue Disease in the Philippines (2000–2011): A Systematic Literature Review
Source: PLoS Negl Trop Dis. 2014 Nov 6;8(11):e3027. doi: 10.1371/journal.pntd.0003027 (PMC4222740; doi:10.1371/journal.pntd.0003027)
Supplement: Table S1 — Evidence table for citations fulfilling the inclusion and exclusion criteria for the literature review (n = 34). (PDF) [file pntd.0003027.s001.pdf]

Table S1. Evidence table for citations fulfilling the inclusion and exclusion criteria for the literature review (n=34).

Citation	Region/geographical area	Study design	Data period: Date range/year	If study:		Summary of data presentation or results/conclusion
				Patients/ population studied (n) [m/f]	Age range	
Ooi et al., 2008 (3)	Nationwide data	Review of surveillance systems	2000–2005			Includes incidence of dengue fever/dengue hemorrhagic fever in South-East Asian countries from 2000 to 2005 (WHO DengueNet) and a subjective evaluation of the status and efficiency of the surveillance systems.
DOH, 2005–2010 (5)	Nationwide data	DoH report	1980–2001, 2004			Presentation of Philippine Health Statistics showing trends in dengue morbidity and case-fatality (1980–2001 and 2004) highlighting the cyclical nature of the disease.
WHO, 2008 (6)	Nationwide data	WHO report	1991–2004			Information from the WHO South-East Asia and Western Pacific regions providing a single source of information on public health issues across the Asia Pacific Region. In a review of the epidemiology of dengue in the Region, the book provides data on the number of reported dengue cases and case-fatality rates in Philippines.
Beatty et al., 2010 (13)	Nationwide data	Working party assessment of dengue surveillance in 22 countries	2005 and 2006			Two Dengue Prevention Boards convened by Pediatric Dengue Vaccine Initiative to discuss dengue surveillance in 22 countries. Philippines' surveillance data presented for 2005/6. Assesses strengths and weaknesses of surveillance for the countries represented and suggested best practice in endemic countries.
Lee Suy, 2008 (16)	Nationwide data	WHO report	1993–2007			The number of cases has been increased to a total of over 55 000 cases in 2007. The case-fatality rate has been maintained at approximately 1% (the CFR in 2007 was 0.9%). Children between 1 and 10 years old are most affected by dengue infection, and infection is essentially equal between males and females. All serotypes of the disease are transmitted in the Philippines.
WHO, 2009 (20)	Nationwide data	WHO report	2006 and 2008			A profile of health in WPRO countries. In 2006, dengue was the tenth leading cause of in-patient morbidity in the Philippines. Report suggests a resurgence in the number of dengue cases early in 2008.

Citation	Region/geographical area	Study design	Data period: Date range/year	If study:		Summary of data presentation or results/conclusion
				Patients/ population studied (n) [m/f]	Age range	
WHO, 2012 (21)	Nationwide data	WHO report	2005–2007			Data on current climate-sensitive disease burdens. Reported dengue cases by month and by morbidity week.
DOH, 2000–2005 (25)	Nationwide data, all regions	Department of Health National and Regional Health Statistics	2000–2005	Total population of the country	All	Data tables
DOH, 2011 (26)	Nationwide data	DOH report	1991–2005			Data tables: leading causes of morbidity in the Philippines.
DOH, 2011 (27)	Nationwide data	DOH report	2001–2006			Data tables presenting leading causes of child mortality.
DOH, 2011 (28)	NCR, CAR, Regions 1-12, CARAGA, ARMM, Foreign	DOH report	2001–2006			Data tables: selected causes of morbidity in the Philippines, by region.
FHSIS, 2000–2009 (29)	Nationwide data	DOH report	2001–2009	6614 [3560:3054]	<1 – ≥65 years	The Field Health Services Information System (FHSIS) provides the Department of Health (DOH) with health-services data to monitor activities on a routine basis (monthly, quarterly or annually) from the Barangay Health Stations, municipality, province, cities and regions.
WHO, 2010 (30)	Nationwide data	WHO surveillance report	1 January 2010 – 18 December 2010			Regular dengue situation updates made available by the WHO WPRO based on officially reported national surveillance data.
WHO, 2011 (31)	Nationwide data	WHO emerging disease surveillance and response	January 2011 – December 2011			Regular dengue situation updates made available by the WHO WPRO based on officially reported national surveillance data.
WHO, 2012 (32)	Nationwide data	WHO surveillance report	2009			In 2009, there were 242,424 dengue cases and 785 dengue deaths were reported in 25 out of 37 countries and territories in the WPRO region: Philippines (57,819 cases, 548 deaths).
Arima et al., 2011 (33)	Nationwide data	WHO summary of Official dengue surveillance data: Suspected or probable dengue cases were reported through the Philippines Integrated Disease Surveillance and Response System	2006–2010	172,288 cases [52.3:47.7]		Dengue surveillance data for 2010 reported a total of 135355 dengue cases, of which 793 were fatal, with a peak (n = 30 009) during the month of August. While all four serotypes circulated, the predominant serotype identified was DENV-3.

Citation	Region/geographical area	Study design	Data period: Date range/year	If study:		Summary of data presentation or results/conclusion
				Patients/ population studied (n) [m/f]	Age range	
NSCB, 2001–2008 (34)	Nationwide data	National Statistical Coordination Board (NSCB) data	2001–2008			Notifiable diseases reported cases by cause 2001–2008 (data reported only reflect cases seen in government health centers, and thus are under-reported).
Contreras et al., 2011 (35)	Quezon City hospital	Observational study: description of DHF patients admitted to the Paediatric Intensive Care Unit (PICU)	January 2005 – December 2009	129 patients [1:1]	Mean age 8.8 years ± 3.4 years	2006 had the highest DHF PICU admissions (41.86%), the lowest in 2005 (4.65%); 60% were in the 6–11 years-old age group; male to female ratio was 1:1. Majority of cases were from Quezon City (37%), Rizal (23%), Metro Manila (9%), Bulacan (5%), Antipolo (4%) and Caloocan (3%).
DOH, 2011 (36)	Nationwide data, split by 17 regions	DOH surveillance report	2010 and 2011			Disease Surveillance Report: 70,204 dengue cases reported by nationwide disease reporting units from January 1 to September 10, 2011. Most of the cases were from: National Capital Region (21.97%), Region III (19.01%), Region IV-A (14.55%).
Edillo et al., 2008 (37)	Rural Guba, Cebu City	Surveillance study: analysis of dengue cases and deaths recorded at sentinel hospitals of the DOH Regional Epidemiological Surveillance Unit, and pupal survey	1997–2008	33771		17,675 dengue cases and 442 deaths were reported in the municipalities or cities of Cebu Province (2002–2007). Cebu City had the highest reported dengue cases throughout this period (range per year = 605–1,670 cases and 8–57 deaths). Both genders were almost equally represented.
Edillo et al., 2009 (38)	Cebu	Surveillance study: analysis of all dengue cases recorded at different Regional and Surveillance Unit sentinel hospitals of the Department of Health, Central Visayas (Region 7)	2002–2007	17675		Year-to-year differences of dengue cases, deaths and case–fatality rates (CFR=1.19–3.73%) were significant. Dengue cases and deaths increased in the rainy season. Children age 1–5 years (31.7%) followed by those age 6–10 years (30.59%) were the most affected.
NDRRMC Update, 2011 (39)	Calabarzon region: Provinces of Cavite, Laguna, Batangas, Rizal and Quezon	Summary report of Regional Disaster Risk Reduction and Management Council (RDRRMC) IV-A conducted a meeting to assess the current dengue situation in Calabarzon	January – August 2011			From January to the first week of August 2011, there were 6,778 cases of dengue with 33 deaths recorded in the Provinces of Cavite, Laguna, Batangas, Rizal and Quezon. Dengue cases concentrated and clustered in identified breeding places of dengue-carrying mosquitoes such as junk shops, vulcanizing shops, tyre collections, etc.

Citation	Region/geographical area	Study design	Data period: Date range/year	If study: Patients/ population studied (n) [m/f]	If study: Age range	Summary of data presentation or results/conclusion
Su et al., 2008 (40)	Metro Manila (also called National Capital Region):16 surrounding cities and municipalities	Retrospective study using National Epidemiology Sentinel Surveillance System incidence data and climatic data from the Philippine Atmospheric, Geophysical and Astronomical Services (PAGASA, Quezon City).	1996–2005			Dengue incidence in Metro Manila was influenced by changing rainfall patterns. Temperature was not significantly related to dengue incidence. This study suggests that surveillance and control of mosquitoes must be intensified during periods of high rainfall.
DOH, 1999–2011 (41)	Nationwide data	Research conducted at NEC Library of the DOH	1999–2011			Research conducted at: NEC Library of the DOH (formerly FETP Library), Philippines. Data analyses provided between 28 May and 20 July 2012.
Lim et al., 2010 (42)	Muntinlupa	Retrospective, descriptive study: assessment of clinico-demographic profile of paediatric patients admitted for dengue at the Research Institute for Tropical Medicine (RITM)	2000–2004	200 qualified for the study [1:1]	Eligible: <19 years. Mean age±SD: 10±4 years. Range: 4 months–18 years. Majority (64%) 6–12 years	51% of the patients had grade II DHF; 49% had acute secondary dengue infection; 32% had recent secondary dengue infection; only 13% had acute primary dengue infection. There was no significant relationship between the severity of dengue infection (WHO Grade) and primary or secondary infection status (r = 0.018, p value = 0.025).
Ypil-Butac et al., 2011 (43)	San Lazaro Hospital (a tertiary infectious disease referral hospital) in Manila	Sero-epidemiologic profiling of patients admitted with dengue-like illness. Outcomes reported: clinical presentation; disease severity; primary vs secondary infections; age range	1 September 2005 – 17 January 2006	275 screened; 104 enrolled; 90 (87%) confirmed dengue [62%:38%]	Mean age, 18 years	6 (7%) primary and 84 (93%) secondary dengue virus infections; 40 (38%) female, 64 (62%) male; mean age 18 years (50% of cases between 13–20 years), only 3% were <7 years and 1 % >35 years. All four serotypes were identified; DENV-3 was predominant.
Capeding et al., 2010 (44)	San Pablo, Laguna, a semi-urban community south of metro Manila	Fever surveillance in a prospective cohort study. Outcomes reported: Dengue incidence, disease severity, primary vs secondary infection, and dengue serotype (RT-PCR)	January 2007 – May 2009	4441 infants	Healthy infants aged 6-18 weeks at enrollment	Age-specific incidence of symptomatic DENV infection: 0.7–2.8/1,000 (2–15 months; mode 8 months). Age-specific incidence (3–8 months) of infant DHF: 0.5/1,000; no DHF cases in infants ≥ 9 months. Incidence rate of unapparent infant infections 103/1,000 person–years: a 6-fold higher rate than symptomatic infant DENV infections.

Citation	Region/geographical area	Study design	Data period: Date range/year	If study:		Summary of data presentation or results/conclusion
				Patients/ population studied (n) [m/f]	Age range	
Bomasang et al., 2008 (45)	Cardinal Santos Medical Center, Greenhills, San Juan	Prospective cohort study 10-month, prospective cohort study conducted in a tertiary referral hospital (Cardinal Santos Medical Center)	November 2006 – August 2007	42 adults clinically suspected to have dengue infection based on the WHO criteria [18:24]	≥18 years	90% of clinically suspected dengue cases confirmed by serologic and virologic examinations. Predominant serotypes were DENV-2 and -3; a trend towards a more severe dengue infection with these serotypes.
Libraty et al., 2009 (46)	San Pablo, Laguna, Philippines	A prospective nested case-control study of DENV infections during infancy to measure associations between maternally derived anti-DENV3 antibody-neutralizing and -enhancing capacities at the time of DENV3 infection and development of infant DHF	January 2007 – January 2008	4441 infants [2276:2165]	2.2 months (0.8–5.6 months)	The study captured 60 infants with DENV infections across a wide spectrum of disease severity. DENV-3 was the predominant serotype among the infants with symptomatic (35/40) and unapparent (15/20) DENV infections, and 59/60 infants had a primary DENV infection.
Carlos et al., 2005 (47)	St. Luke's Medical Center in Quezon City	Prospective study to determine differences in the clinical features and hematologic abnormalities between DF and DHF. Outcomes reported: disease severity, clinical features, hematologic abnormalities, dengue serotype (RT-PCR)	January 1999 and December 2001	503 subjects screened: 359 patients with dengue (1.49:1)	2–17 years	Of the 503 subjects screened, 359 were confirmed as having a dengue-virus infection: one third was diagnosed with DHF. Coagulation abnormalities associated with most DHF patients were thrombocytopenia and increased fibrinolysis.
Oishi et al., 2006 (48)	St. Luke's Medical Center (SLMC), Quezon City, Metro Manila, Philippines	Prospective study to define the clinical and hematological differences between DF/DHF and other febrile illnesses	January 1999 – December 2001	503 hospitalized pediatric patients with acute febrile illness, of which 359 (71.4%) were diagnosed DF/DFH. [1.5:1]	Eligible: 2-17 years. Mean±SD age was 9.9±4.2 years for the 239 DF patients and 9.8±3.7 years for the 120 DHF patients	359 cases (71.4%) were diagnosed with a dengue-virus infection. Most of the cases with dengue virus infection were associated with DENV-1 and DENV-2. One third of the patients with dengue virus infections had DHF, most without shock. A low incidence of dengue shock syndrome and a low fatality rate among pediatric patients with dengue virus infection was found.

Citation	Region/geographical area	Study design	Data period: Date range/year	If study:		Summary of data presentation or results/conclusion
				Patients/ population studied (n) [m/f]	Age range	
Alera et al., 2009 (49)	Cebu City	Cross-sectional, tertiary, hospital-based passive surveillance study	January – December 2008	176 enrolled; virologically confirmed 154 [1.2:1]	Overall: 2–32 years; majority (96%) <15 years	Acute secondary infections: 144 (94%); acute primary infections: 6 (4%). Clinical diagnosis of laboratory confirmed dengue cases: 69 (45%) DHF Group 2, 43 (28%) DHF Group 1, 33 (21%) DSS (DHF Group 3/4), 5 (3%) DF, and 4 (3%) systemic viral infection. Circulating serotypes: DENV-1: 10 (12%); DENV-2: 9 (11%); DENV-3: 65 (76%); DENV-4: 1 (1%)..
Salda et al., 2005 (50)	Nationwide	Molecular epidemiology study: determination of DENV-2 genetic variants circulating locally and identification of possible associations with severity of clinical presentation. Outcomes reported: phylogenetic analysis of dengue virus serotype 2 isolates	1995 – 2002	Samples consisted of 41 DENV-2 isolates from outbreaks and epidemics		Phylogenetic analysis revealed two distinct circulating genotypes (Asian 2 and Cosmopolitan) with the potential to cause severe hemorrhagic disease. The Cosmopolitan genotype has gradually replaced Asian genotype 2 in the Philippines. Members of this genotype were closely related to viruses from Australia, Singapore and Thailand.
Destura et al., 2010 (51)	N/A	Gene sequence analysis of 14 Philippine DENV-3 isolates compared with 17 other geographically diverse DENV-3 isolates	2008 – 2010	N/A		The study clearly revealed a different subtype compared with other reported sequences of DENV-3 viruses. The Philippine isolates did not fall into any of the groups of the reported genotypes suggesting the identification of a new genotype.
