# Supplementary material for: Epidemiology of Dengue Disease in the Philippines (2000–2011): A Systematic Literature Review
Source: PLoS Negl Trop Dis. 2014 Nov 6;8(11):e3027. doi: 10.1371/journal.pntd.0003027 (PMC4222740; doi:10.1371/journal.pntd.0003027)
Supplement: Table S2 — Number and incidence of dengue disease cases in the Philippines: national data. DF, dengue fever; DHF, dengue haemorrhagic fever; DoH, Department of Health; DSS, dengue shock syndrome; FHSIS, Field Health Surveillance Information System; NSCB, National Statistical Coordination Board; WHO, World Health Organization. *Values estimated from graphs. (PDF) [file pntd.0003027.s002.pdf]

Table S2. Number and incidence of dengue disease cases in the Philippines: national data.

Year	Cases reported (n)			Incidence (per 100,000 population)			Source of data. First author, year [Ref]
	DF	DHF/DSS	All	DF	DHF/DSS	All	
2000			6614			9	DoH 2000–2005 [25]
2000						9*	DoH 2005–2010 [5]
2000			6614			8.7	FHSIS 2000–2009 [29]
2000			8000				WHO 2008 [6]
2000			8561				WHO 2008 [16]
2001			23,235			30	DoH 2000–2005 [25]
2001						30*	DoH 2005–2010 [5]
2001			23,235			29.8	FHSIS 2000–2009 [29]
2001			23,235				NSCB 2001–2008 [34]
2001			25,216				WHO 2008 [6]
2001			25,002				WHO 2008 [16]
2002			13,187			16.6	DoH 2000–2005 [25]
2002			13,187			16.6	FHSIS 2000–2009 [29]
2002			13,187				NSCB 2001–2008 [34]
2002			16,500				WHO 2008 [6]
2002			16,663				WHO 2008 [16]
2003			22,789			28.1	DoH 2000–2005 [25]
2003	18,039	4750	22,789	23.0	6.1		FHSIS 2000–2009 [29]
2003			18,039				NSCB 2001–2008 [34]
2003			29,000				WHO 2008 [6]
2003			29,946				WHO 2008 [16]
2004						13	DoH 2005–2010 [5]
2004			15,838			19	DoH 2000–2005 [25]
2004			15,838			19.0	DoH 2011 [26]
2004			15,838			19.8	FHSIS 2000–2009 [29]
2004			15,838				NSCB 2001–2008 [34]
2004			23,000				WHO 2008 [6]
2004			23,040				WHO 2008 [16]
2005			20,107			23.6	DoH 2011 [26]
2005			20,107			24	DoH 2000–2005 [25]
2005		4092	20,107		4.9	24.1	FHSIS 2000–2009 [29]
2005			4092				NSCB 2001–2008 [34]
2005			33,901				WHO 2008 [16]
2005			20,107			24.1	WHO 2012 [21]
2005			33,490				Beatty 2010 [13]
2006		14,310	15,279		17.7	19.6	FHSIS 2000–2009 [29]
2006			15,279			19.60	WHO 2009 [20]
2006			37,101				WHO 2008 [16]

Year	Cases reported (n)			Incidence (per 100,000 population)			Source of data. First author, year [Ref]
	DF	DHF/DSS	All	DF	DHF/DSS	All	
2006			37,101				Arima 2011 [33]
2006			36,891				Beatty 2010 [13]
2007		11,915	23,773		14.1	28.2	FHSIS 2000–2009 [29]
2007			11,915				NSCB 2001–2008 [34]
2007			55,135				WHO 2008 [16]
2007			55,639				Arima 2011 [33]
2008		13,014			14.5		FHSIS 2000–2009 [29]
2008			13,014				NSCB 2001–2008 [34]
2008			39,620				WHO 2009 [20]
2008			39,620				Arima 2011 [33]
2009		5617			6.2		FHSIS 2000–2009 [29]
2009			56,545				WHO 2011 [30]
2009			57,819				WHO 2012 [32]
2009			57,819				Arima 2011 [33]
2010			173,033*				WHO 2012 [31]
2010			131,976				WHO 2011 [30]
2010			135,355			144.55	Arima 2011 [33]
2011			118,868*				WHO 2012 [31]

DF, dengue fever; DHF, dengue haemorrhagic fever; DoH, Department of Health; DSS, dengue shock syndrome; FHSIS, Field Health Surveillance Information System; NSCB, National Statistical Coordination Board; WHO, World Health Organization.

*****Values estimated from graphs.
