# Supplementary material for: Epidemiology of Dengue Disease in the Philippines (2000–2011): A Systematic Literature Review
Source: PLoS Negl Trop Dis. 2014 Nov 6;8(11):e3027. doi: 10.1371/journal.pntd.0003027 (PMC4222740; doi:10.1371/journal.pntd.0003027)
Supplement: Table S3 — Dengue disease-related deaths and case fatality rate in the Philippines: national data. CFR, case fatality rate; DF, dengue fever; DHF, dengue haemorrhagic fever; DoH, Department of Health; DSS, dengue shock syndrome; WHO, World Health Organization. *Values estimated from graphs. †Cases in children (5–9 years old only). ‡Cases in children (10–14 years old only). (PDF) [file pntd.0003027.s003.pdf]

Table S3. Dengue disease-related deaths and case fatality rate in the Philippines: national data.

Year	Death cases (n)			CFR (per 100 cases)			Source of data. First author, year [Ref]
	DF	DHF/DSS	All	DF	DHF/DSS	All	
2000						0.5%*	DoH 2005–2010 [5]
2000			386				DoH 2000–2005 [25]
2000						0.9%	WHO 2008 [6]
2000						0.8%	WHO 2008 [16]
2001			641				DoH 2000–2005 [25]
2001			214†				DoH 2011 [27]
2001			177			0.75%	WHO 2008 [6]
2001						0.7%	WHO 2008 [16]
2002			576				DoH 2011 [28]
2002			576				DoH 2000–2005 [25]
2002						1.1%	WHO 2008 [6]
2002						1.1%	WHO 2008 [16]
2003			831				DoH 2011 [28]
2003			831				DoH 2000–2005 [25]
2003			282†				DoH 2011 [27]
2003						0.8%	WHO 2008 [6]
2003						0.8%	WHO 2008 [16]
2004			761				DoH 2011 [28]
2004						1.7%	DoH 2005–2010 [5]
2004			761				DoH 2000–2005 [25]
2004			256†				DoH 2011 [27]
2004						1.1%	WHO 2008 [6]
2004						1.0%	WHO 2008 [16]
2005			887				DoH 2011 [28]
2005			887				DoH 2000–2005 [25]
2005			320†				DoH 2011 [27]
2005						1.2%	WHO 2008 [16]
2006			1017				DoH 2011 [28]
2006			349†				DoH 2011 [27]
2006			131‡				DoH 2011 [27]
2006						1.0%	WHO 2008 [16]
2006			378			1.02%	Arima 2011 [33]
2007						0.9%	WHO 2008 [16]
2007			533			0.96%	Arima 2011 [33]
2008			373				WHO 2009 [20]
2008			373			0.94%	Arima 2011 [33]
2009			548				WHO 2012 [32]
2009			548			0.95%	Arima 2011 [33]
2010			788			0.6%	WHO 2011 [30]
2010			793			0.94%	Arima 2011 [33]
2011			639*			0.54%*	WHO 2012 [31]

CFR, case fatality rate; DF, dengue fever; DHF, dengue haemorrhagic fever; DoH, Department of Health; DSS, dengue shock syndrome; WHO, World Health Organization.

*Values estimated from graphs.
†Cases in children (5–9 years old only).
‡Cases in children (10–14 years old only).
