# Supplementary material for: Epidemiology of Dengue Disease in the Philippines (2000–2011): A Systematic Literature Review
Source: PLoS Negl Trop Dis. 2014 Nov 6;8(11):e3027. doi: 10.1371/journal.pntd.0003027 (PMC4222740; doi:10.1371/journal.pntd.0003027)
Supplement: Table S4 — Number and incidence of dengue disease in the Philippines. (A) Regional data; (B) outbreak data from Field Epidemiology Training Program Fellows' reports; (C) outbreak data from Regional Surveillance Units reports. ARMM, Autonomous Region in Muslim Mindanao; CAR, Cordillera Administrative Region; CFR, case fatality rate; DF, dengue fever; DoH, Department of Health; FHSIS, Field Health Service Information System; NCR, National Capital Region; NDRRMC, National Disaster Risk Reduction and Management Council. *1 January to 10 September, 2010 or 2011. †January to August, 2011. ‡Ongoing outbreak. (PDF) [file pntd.0003027.s004.pdf]

Table S4. Number and incidence of dengue disease in the Philippines.

(A) Regional data; (B) outbreak data from Field Epidemiology Training Program Fellows' reports; (C) outbreak data from Regional Surveillance Units reports.

Year	Region	DF		DF mortality		Source of data. First author, year [Ref]
		n	Incidence (per 100,000 population)	n	CFR (%)	
**(A) Regional data**						
1996–2005	NCR		0.367–20.358			Su 2008 [40]
1997–2008	Rural Guba, Cebu City (Region VII)	33,771		831		Edillo 2009 [38]
2000	NCR	2565	25	111	1.1	DoH 2000–2005 [25]
2000	NCR	2565	24.7			FHSIS 2000–2009 [29]
2000	CAR	125	9	5	0.4	DoH 2000–2005 [25]
2000	CAR	125	8.9			FHSIS 2000–2009 [29]
2000	Region I	316	8	20	0.5	DoH 2000–2005 [25]
2000	Region I	316	7.6			FHSIS 2000–2009 [29]
2000	Region II	127	5	7	0.2	DoH 2000–2005 [25]
2000	Region II	127	4.5			FHSIS 2000–2009 [29]
2000	Region III	740	10	42	0.5	DoH 2000–2005 [25]
2000	Region III	740	9.6			FHSIS 2000–2009 [29]
2000	Region IV	947	8	70	0.6	DoH 2000–2005 [25]
2000	Region IV	947	8.4			FHSIS 2000–2009 [29]
2000	Region V	335	7	14	0.3	DoH 2000–2005 [25]
2000	Region V	335	7			FHSIS 2000–2009 [29]
2000	Region VI	72	1	14	0.2	DoH 2000–2005 [25]

Year	Region	DF		DF mortality		Source of data. First author, year [Ref]
		n	Incidence (per 100,000 population)	n	CFR (%)	
2000	Region VI	72	1.1			FHSIS 2000–2009 [29]
2000	Region VII	464	8	49	0.9	DoH 2000–2005 [25]
2000	Region VII	464	8.4			FHSIS 2000–2009 [29]
2000	Region VIII	242	7	10	0.3	DoH 2000–2005 [25]
2000	Region VIII	242	6.5			FHSIS 2000–2009 [29]
2000	Region IX	161	5	6	0.2	DoH 2000–2005 [25]
2000	Region IX	161	5.1			FHSIS 2000–2009 [29]
2000	Region X	92	3	9	0.3	DoH 2000–2005 [25]
2000	Region X	92	3.3			FHSIS 2000–2009 [29]
2000	Region XI	55	1	15	0.3	DoH 2000–2005 [25]
2000	Region XI	55	1.1			FHSIS 2000–2009 [29]
2000	Region XII	335	13	10	0.4	DoH 2000–2005 [25]
2000	Region XII	335	12.6			FHSIS 2000–2009 [29]
2000	Region XIII	21	1	4	0.2	DoH 2000–2005 [25]
2000	Region XIII	21	1			FHSIS 2000–2009 [29]
2000	ARMM	17	1	0	0	DoH 2000–2005 [25]
2000	ARMM	17	0.8			FHSIS 2000–2009 [29]
2001	NCR			121		DoH 2011 [28]
2001	NCR	4941	47	121	1.1	DoH 2000–2005 [25]
2001	NCR	4941	46.7			FHSIS 2000–2009 [29]
2001	CAR			9		DoH 2011 [28]

Year	Region	DF		DF mortality		Source of data. First author, year [Ref]
		n	Incidence (per 100,000 population)	n	CFR (%)	
2001	CAR	1555	108	9	0.6	DoH 2000–2005 [25]
2001	CAR	1555	108.4			FHSIS 2000–2009 [29]
2001	Region I			36		DoH 2011 [28]
2001	Region I	739	18	36	0.9	DoH 2000–2005 [25]
2001	Region I	739	17.5			FHSIS 2000–2009 [29]
2001	Region II			14		DoH 2011 [28]
2001	Region II	791	28	14	0.5	DoH 2000–2005 [25]
2001	Region II	791	27.7			FHSIS 2000–2009 [29]
2001	Region III			65		DoH 2011 [28]
2001	Region III	2566	33	65	0.8	DoH 2000–2005 [25]
2001	Region III	2566	32.7			FHSIS 2000–2009 [29]
2001	Region IV			119		DoH 2011 [28]
2001	Region IV	1833	16	119	1	DoH 2000–2005 [25]
2001	Region IV	1833	15.8			FHSIS 2000–2009 [29]
2001	Region V			23		DoH 2011 [28]
2001	Region V	614	13	23	0.5	DoH 2000–2005 [25]
2001	Region V	614	12.7			FHSIS 2000–2009 [29]
2001	Region VI			18		DoH 2011 [28]
2001	Region VI	2220	35	18	0.3	DoH 2000–2005 [25]
2001	Region VI	2220	34.5			FHSIS 2000–2009 [29]
2001	Region VII			114		DoH 2011 [28]
2001	Region VII	2512	45	114	2	DoH 2000–2005 [25]
2001	Region VII	2512	44.5			FHSIS 2000–2009 [29]
2001	Region VIII			21		DoH 2011 [28]

Year	Region	DF		DF mortality		Source of data. First author, year [Ref]
		n	Incidence (per 100,000 population)	n	CFR (%)	
2001	Region VIII	1558	41	21	0.5	DoH 2000–2005 [25]
2001	Region VIII	1558	40.7			FHSIS 2000–2009 [29]
2001	Region IX			13		DoH 2011 [28]
2001	Region IX	987	31	13	0.4	DoH 2000–2005 [25]
2001	Region IX	987	30.6			FHSIS 2000–2009 [29]
2001	Region X			15		DoH 2011 [28]
2001	Region X	1712	60	15	0.5	DoH 2000–2005 [25]
2001	Region X	1712	60.4			FHSIS 2000–2009 [29]
2001	Region XI			45		DoH 2011 [25]
2001	Region XI	75	1	45	0.8	DoH 2000–2005 [25]
2001	Region XI	75	1.4			FHSIS 2000–2009 [29]
2001	Region XII			12		DoH 2011 [28]
2001	Region XII	767	28	12	0.4	DoH 2000–2005 [25]
2001	Region XII	767	28.2			FHSIS 2000–2009 [29]
2001	Region XIII			13		DoH 2011 [28]
2001	Region XIII	295	13	13	0.6	DoH 2000–2005 [25]
2001	Region XIII	295	13.2			FHSIS 2000–2009 [29]
2001	ARMM			3		DoH 2011 [28]
2001	ARMM	70	3	3	0.1	DoH 2000–2005 [25]
2001	ARMM	70	3.1			FHSIS 2000–2009 [29]
2002–2007	Cebu (Region VII)	17,675		442		Edillo 2008 [37]
2002	NCR			88		DoH 2011 [28]
2002	NCR	2526	24	88	0.8	DoH 2000–2005 [25]
2002	NCR	2526	23.5			FHSIS 2000–2009 [29]
2002	CAR			3		DoH 2011 [28]

Year	Region	DF		DF mortality		Source of data. First author, year [Ref]
		n	Incidence (per 100,000 population)	n	CFR (%)	
2002	CAR	104	7	3	0.2	DoH 2000–2005 [25]
2002	CAR	104	7.1			FHSIS 2000–2009 [29]
2002	Region I			16		DoH 2011 [28]
2002	Region I	81	2	16	0.4	DoH 2000–2005 [25]
2002	Region I	81	1.9			FHSIS 2000–2009 [29]
2002	Region II			12		DoH 2011 [28]
2002	Region II	862	30	12	0.4	DoH 2000–2005 [25]
2002	Region II	862	29.6			FHSIS 2000–2009 [29]
2002	Region III			22		DoH 2011 [28]
2002	Region III	598	7	22	0.3	DoH 2000–2005 [25]
2002	Region III	598	7.3			FHSIS 2000–2009 [29]
2002	Region IV			85		DoH 2011 [28]
2002	Region IV	1562	13	85	0.7	DoH 2000–2005 [25]
2002	Region IV	1562	13.3			FHSIS 2000–2009 [29]
2002	Region V			36		DoH 2011 [28]
2002	Region V	521	11	36	0.7	DoH 2000–2005 [25]
2002	Region V	521	10.6			FHSIS 2000–2009 [29]
2002	Region VI			29		DoH 2011 [28]
2002	Region VI	2618	40	29	0.4	DoH 2000–2005 [25]
2002	Region VI	2618	40.0			FHSIS 2000–2009 [29]
2002	Region VII			107		DoH 2011 [28]
2002	Region VII	744	13	107	1.9	DoH 2000–2005 [25]
2002	Region VII	744	12.9			FHSIS 2000–2009 [29]
2002	Region VIII			20		DoH 2011 [28]

Year	Region	DF		DF mortality		Source of data. First author, year [Ref]
		n	Incidence (per 100,000 population)	n	CFR (%)	
2002	Region VIII	922	24	20	0.5	DoH 2000–2005 [25]
2002	Region VIII	922	23.6			FHSIS 2000–2009 [29]
2002	Region IX			10		DoH 2011 [28]
2002	Region IX	623	21	10	0.3	DoH 2000–2005 [25]
2002	Region IX	623	20.5			FHSIS 2000–2009 [29]
2002	Region X			26		DoH 2011 [28]
2002	Region X	796	28	26	0.9	DoH 2000–2005 [25]
2002	Region X	796	27.5			FHSIS 2000–2009 [29]
2002	Region XI			98		DoH 2011 [28]
2002	Region XI	516	9	98	1.8	DoH 2000–2005 [25]
2002	Region XI	516	9.4			FHSIS 2000–2009 [29]
2002	Region XII			8		DoH 2011 [28]
2002	Region XII	483	17	8	0.3	DoH 2000–2005 [25]
2002	Region XII	483	17.3			FHSIS 2000–2009 [29]
2002	Region XIII			15		DoH 2011 [28]
2002	Region XIII	213	9	15	0.7	DoH 2000–2005 [25]
2002	Region XIII	213	9.3			FHSIS 2000–2009 [29]
2002	ARMM			1		DoH 2011 [28]
2002	ARMM	18	1	1	0	DoH 2000–2005 [25]
2002	ARMM	18	0.8			FHSIS 2000–2009 [29]
2003	NCR			148		DoH 2011 [28]
2003	NCR	3001	28	9	0.6	DoH 2000–2005 [25]
2003	NCR	3017	27.6			FHSIS 2000–2009 [29]
2003	CAR			9		DoH 2011 [28]

Year	Region	DF		DF mortality		Source of data. First author, year [Ref]
		n	Incidence (per 100,000 population)	n	CFR (%)	
2003	CAR		0	30	0.7	DoH 2000–2005 [25]
2003	Region I			30		DoH 2011 [28]
2003	Region I	874	20	13	0.4	DoH 2000–2005 [25]
2003	Region I	874	20.1			FHSIS 2000–2009 [29]
2003	Region II			13		DoH 2011 [28]
2003	Region II	1060	36	66	0.8	DoH 2000–2005 [25]
2003	Region II	1044	35.2			FHSIS 2000–2009 [29]
2003	Region III			66		DoH 2011 [28]
2003	Region III	1882	23	100	1	DoH 2000–2005 [25]
2003	Region III	1882	22.6			FHSIS 2000–2009 [29]
2003	Region IV			100		DoH 2011 [28]
2003	Region IV-A	1757	18	16	0.7	DoH 2000–2005 [25]
2003	Region IV-A	1757	18.2			FHSIS 2000–2009 [29]
2003	Region IV-B	221	9	43	0.9	DoH 2000–2005 [25]
2003	Region IV-B	221	9.2			FHSIS 2000–2009 [29]
2003	Region V			16		DoH 2011 [28]
2003	Region V	546	11	18	0.3	DoH 2000–2005 [25]
2003	Region V	546	10.9			FHSIS 2000–2009 [29]
2003	Region VI			43		DoH 2011 [28]
2003	Region VI	779	12	100	1.7	DoH 2000–2005 [25]
2003	Region VI	779	11.7			FHSIS 2000–2009 [29]
2003	Region VII			18		DoH 2011 [28]
2003	Region VII	1555	27	21	0.5	DoH 2000–2005 [25]
2003	Region VII	1555	26.5			FHSIS 2000–2009 [29]

Year	Region	DF		DF mortality		Source of data. First author, year [Ref]
		n	Incidence (per 100,000 population)	n	CFR (%)	
2003	Region VIII			100		DoH 2011 [28]
2003	Region VIII	1008	25	9	0.3	DoH 2000–2005 [25]
2003	Region VIII	1008	25.3			FHSIS 2000–2009 [29]
2003	Region IX			21		DoH 2011 [28]
2003	Region IX	841	28	61	1.6	DoH 2000–2005 [25]
2003	Region IX	841	27.0			FHSIS 2000–2009 [29]
2003	Region X			9		DoH 2011 [28]
2003	Region X	321	8	109	2.8	DoH 2000–2005 [25]
2003	Region X	321	8.4			FHSIS 2000–2009 [29]
2003	Region XI			61		DoH 2011 [28]
2003	Region XI	3464	88	61	1.8	DoH 2000–2005 [25]
2003	Region XI	3464	88.1			FHSIS 2000–2009 [29]
2003	Region XII			109		DoH 2011 [28]
2003	Region XII	614	18	21	0.9	DoH 2000–2005 [25]
2003	Region XII	614	17.2			FHSIS 2000–2009 [29]
2003	Region XIII			61		DoH 2011 [28]
2003	Region XIII	116	5	6	0.2	DoH 2000–2005 [25]
2003	Region XIII	116	5.0			FHSIS 2000–2009 [29]
2003	ARMM			21		DoH 2011 [28]
2003	ARMM			9	0.6	DoH 2000–2005 [25]
2003	Foreign			6		DoH 2011 [28]
2004	NCR			131		DoH 2011 [28]
2004	NCR	1957	18	131	1.2	DoH 2000–2005 [25]
2004	NCR	1957	17.7			FHSIS 2000–2009 [29]
2004	CAR			2		DoH 2011 [28]

Year	Region	DF		DF mortality		Source of data. First author, year [Ref]
		n	Incidence (per 100,000 population)	n	CFR (%)	
2004	CAR		0	2	0.1	DoH 2000–2005 [25]
2004	Region I			31		DoH 2011 [28]
2004	Region I	569	12	31	0.7	DoH 2000–2005 [25]
2004	Region II			9		DoH 2011 [28]
2004	Region II	504	17	9	0.3	DoH 2000–2005 [25]
2004	Region III			60		DoH 2011 [28]
2004	Region III	1728	19	60	0.7	DoH 2000–2005 [25]
2004	Region III	1728	20.3			FHSIS 2000–2009 [29]
2004	Region IV			89		DoH 2011 [28]
2004	Region IV-A	848	8	89	0.9	DoH 2000–2005 [25]
2004	Region IV-A	848	8.5			FHSIS 2000–2009 [29]
2004	Region IV-B	111	4	11	0.4	DoH 2000–2005 [25]
2004	Region V			11		DoH 2011 [28]
2004	Region V	767	15	22	0.4	DoH 2000–2005 [25]
2004	Region V	767				FHSIS 2000–2009 [29]
2004	Region VI			22		DoH 2011 [28]
2004	Region VI	12,265	19	32	0.5	DoH 2000–2005 [25]
2004	Region VI	1265	18.5			FHSIS 2000–2009 [29]
2004	Region VI			32		DoH 2011 [28]
2004	Region VI	752	12	59	0.9	DoH 2000–2005 [25]
2004	Region VI	752	12.6			FHSIS 2000–2009 [29]
2004	Region VIII			59		DoH 2011 [28]
2004	Region VIII	969	25	20	0.5	DoH 2000–2005 [25]
2004	Region IX			20		DoH 2011 [28]
2004	Region IX	566	19	16	0.5	DoH 2000–2005 [25]

Year	Region	DF		DF mortality		Source of data. First author, year [Ref]
		n	Incidence (per 100,000 population)	n	CFR (%)	
2004	Region X			16		DoH 2011 [28]
2004	Region X	1256	33	59	1.5	DoH 2000–2005 [25]
2004	Region X	1256	32.2			FHSIS 2000–2009 [29]
2004	Region XI			59		DoH 2011 [28]
2004	Region XI	3891	98	126	3.2	DoH 2000–2005 [25]
2004	Region XI	3891	97.0			FHSIS 2000–2009 [29]
2004	Region XII			126		DoH 2011 [28]
2004	Region XII	993	29	56	1.7	DoH 2000–2005 [25]
2004	Region XII	993	27.2			FHSIS 2000–2009 [29]
2004	Region XIII			56		DoH 2011 [28]
2004	Region XIII	1097	48	36	1.6	DoH 2000–2005 [25]
2004	Region XIII	1097	45.8			FHSIS 2000–2009 [29]
2004	ARMM			36		DoH 2011 [28]
2004	ARMM			2	0.1	DoH 2000–2005 [25]
2004	Foreign			2		DoH 2011 [28]
2005	NCR			185		DoH 2011 [28]
2005	NCR	2102	20	185	1.7	DoH 2000–2005 [25]
2005	NCR	2102	18.7			FHSIS 2000–2009 [29]
2005	CAR			14		DoH 2011 [28]
2005	CAR			14	0.9	DoH 2000–2005 [25]
2005	Region I			34		DoH 2011 [25]
2005	Region I			34	0.7	DoH 2000–2005 [25]
2005	Region II			16		DoH 2011 [28]
2005	Region II			16	0.5	DoH 2000–2005 [25]
2005	Region III			66		DoH 2011 [28]

Year	Region	DF		DF mortality		Source of data. First author, year [Ref]
		n	Incidence (per 100,000 population)	n	CFR (%)	
2005	Region III	2320	25	66	0.7	DoH 2000–2005 [25]
2005	Region III	2320	26.9			FHSIS 2000–2009 [29]
2005	Region IV			78		DoH 2011 [28]
2005	Region IV-A			78	0.7	DoH 2000–2005 [25]
2005	Region IV-B			9	0.3	DoH 2000–2005 [25]
2005	Region IV-B	351	6.8			FHSIS 2000–2009 [29]
2005	Region V			9		DoH 2011 [28]
2005	Region V	351	68	16	0.3	DoH 2000–2005 [25]
2005	Region VI			16		DoH 2011 [28]
2005	Region VI	2270	33	38	0.6	DoH 2000–2005 [25]
2005	Region VII			38		DoH 2011 [28]
2005	Region VII	1500	24	138	2.2	DoH 2000–2005 [25]
2005	Region VII	1500	24.7			FHSIS 2000–2009 [29]
2005	Region VIII			138		DoH 2011 [28]
2005	Region VIII			10	0.2	DoH 2000–2005 [25]
2005	Region IX			10		DoH 2011 [28]
2005	Region IX	2568	84	28	0.9	DoH 2000–2005 [25]
2005	Region IX	2568	78.8			FHSIS 2000–2009 [29]
2005	Region X			28		DoH 2011 [28]
2005	Region X	2792	71	96	2.4	DoH 2000–2005 [25]
2005	Region X	2792	70.5			FHSIS 2000–2009 [29]
2005	Region XI			96		DoH 2011 [28]
2005	Region XI	2814	70	77	1.9	DoH 2000–2005 [25]
2005	Region XI	2814	68.7			FHSIS 2000–2009 [29]
2005	Region XII			77		DoH 2011 [28]

Year	Region	DF		DF mortality		Source of data. First author, year [Ref]
		n	Incidence (per 100,000 population)	n	CFR (%)	
2005	Region XII	3044	88	45	1.3	DoH 2000–2005 [25]
2005	Region XII	3044	81.2			FHSIS 2000–2009 [29]
2005	Region XIII			45		DoH 2011 [28]
2005	Region XIII	622	27	23	1	DoH 2000–2005 [25]
2005	ARMM			23		DoH 2011 [28]
2005	ARMM			14	0.4	DoH 2000–2005 [25]
2005	Foreign			14		DoH 2011 [28]
2005	Quezon City Hospital (NCR)	6				Contreras 2009 [35]
2006	NCR			185		DoH 2011 [28]
2006	NCR	2755	19.6			FHSIS 2000–2009 [29]
2006	CAR			345		DoH 2011 [28]
2006	Region I			7		DoH 2011 [28]
2006	Region I	1002	22			FHSIS 2000–2009 [29]
2006	Region II			46		DoH 2011 [28]
2006	Region II	549	17.6			FHSIS 2000–2009 [29]
2006	Region III			32		DoH 2011 [28]
2006	Region III	2481	37.2			FHSIS 2000–2009 [29]
2006	Region IV			110		DoH 2011 [28]
2006	Region IV-A	1848	17.6			FHSIS 2000–2009 [29]
2006	Region IV-B	255	10.1			FHSIS 2000–2009 [29]
2006	Region V			158		DoH 2011 [28]
2006	Region VI			11		DoH 2011 [28]
2006	Region VII			14		DoH 2011 [28]
2006	Region VII	1681	27.2			FHSIS 2000–2009 [29]
2006	Region VIII			39		DoH 2011 [28]
2006	Region IX			100		DoH 2011 [28]
2006	Region IX	896	26.9			FHSIS 2000–2009 [29]

Year	Region	DF		DF mortality		Source of data. First author, year [Ref]
		n	Incidence (per 100,000 population)	n	CFR (%)	
2006	Region X			12		DoH 2011 [28]
2006	Region XI			17		DoH 2011 [28]
2006	Region XI	997	23.9			FHSIS 2000–2009 [29]
2006	Region XII			42		DoH 2011 [28]
2006	Region XII	1473	38.3			FHSIS 2000–2009 [29]
2006	Region XIII			33		DoH 2011 [28]
2006	ARMM			36		DoH 2011 [28]
2006	Foreign			8		DoH 2011 [28]
2006	Quezon City Hospital (NCR)	84				Contreras 2009 [35]
2007	NCR	3036	26.3			FHSIS 2000–2009 [29]
2007	Region I	705	15.5			FHSIS 2000–2009 [29]
2007	Region III	1388	14.3			FHSIS 2000–2009 [29]
2007	Region IV-A	3037	25.9			FHSIS 2000–2009 [29]
2007	Region VII	4331	67.7			FHSIS 2000–2009 [29]
2007	Region IX	1609	49.8			FHSIS 2000–2009 [29]
2007	Region X	1442	36.5			FHSIS 2000–2009 [29]
2007	Region XI	2380	57.3			FHSIS 2000–2009 [29]
2007	Region XII	2473	64.7			FHSIS 2000–2009 [29]
2007	Region XIII	1097	47.8			FHSIS 2000–2009 [29]
2007	Quezon City Hospital (NCR)	20				Contreras 2009 [35]
2008	NCR	3927	34.9			FHSIS 2000–2009 [29]
2008	Region I	1775	35.7			FHSIS 2000–2009 [29]
2008	Region III	2931	30.0			FHSIS 2000–2009 [29]

Year	Region	DF		DF mortality		Source of data. First author, year [Ref]
		n	Incidence (per 100,000 population)	n	CFR (%)	
2008	Region IV-A	2097	18.4			FHSIS 2000–2009 [29]
2008	Region VII	1284	19.0			FHSIS 2000–2009 [29]
2008	Region X	260	6.2			FHSIS 2000–2009 [29]
2008	Region XI	3671	101.0			FHSIS 2000–2009 [29]
2008	Quezon City Hospital (NCR)	28				Contreras 2009 [35]
2009	NCR	2348	20.6			FHSIS 2000–2009 [29]
2009	CAR	324	19.5			FHSIS 2000–2009 [29]
2009	Region I	226	4.5			FHSIS 2000–2009 [29]
2009	Region IV-A	1510	13.0			FHSIS 2000–2009 [29]
2009	Region VII	547	7.9			FHSIS 2000–2009 [29]
2009	Region VIII	1011	23.2			FHSIS 2000–2009 [29]
2009	Region XI	3799	102.8			FHSIS 2000–2009 [29]
2009	Quezon City Hospital (NCR)	21				Contreras 2009 [35]
2010*	Region I	3451		32	0.93	DoH 2011 [36]
2010*	Region II	1451		17	1.17	DoH 2011 [36]
2010*	Region III	3529		27	0.77	DoH 2011 [36]
2010*	Region IV-A	11,872		50	0.42	DoH 2011 [36]
2010*	Region IV-B	1915		19	0.99	DoH 2011 [36]
2010*	Region V	1630		9	0.55	DoH 2011 [36]
2010*	Region VI	17,593		84	0.48	DoH 2011 [36]
2010*	Region VII	6717		46	0.68	DoH 2011 [36]
2010*	Region VIII	7361		74	1.01	DoH 2011 [36]
2010*	Region IX	2516		34	1.35	DoH 2011 [36]
2010*	Region X	5368		55	1.02	DoH 2011 [36]
2010*	Region XI	6057		47	0.78	DoH 2011 [36]
2010*	Region XII	8293		42	0.51	DoH 2011 [36]
2010*	ARMM	1043		12	1.15	DoH 2011 [36]

Year	Region	DF		DF mortality		Source of data. First author, year [Ref]
		n	Incidence (per 100,000 population)	n	CFR (%)	
2010*	CAR	4074		8	0.2	DoH 2011 [36]
2010*	Region XIII	2113		8	0.38	DoH 2011 [36]
2010*	NCR	9720		56	0.58	DoH 2011 [36]
2010*	Kalookan City (NCR)	1187		5	0.42	DoH 2011 [36]
2010*	Las Pinas City (NCR)	149		2	1.34	DoH 2011 [36]
2010*	Makati City (NCR)	206		0		DoH 2011 [36]
2010*	Malabon City (NCR)	546		3	0.55	DoH 2011 [36]
2010*	Mandaluyong City (NCR)	343		1	0.29	DoH 2011 [36]
2010*	Manila City (NCR)	1374		9	0.66	DoH 2011 [36]
2010*	Marikina City (NCR)	368		2	0.54	DoH 2011 [36]
2010*	Muntinlupa City (NCR)	203		0		DoH 2011 [36]
2010*	Navotas City (NCR)	497		3	0.6	DoH 2011 [36]
2010*	Paranaque City (NCR)	418		0		DoH 2011 [36]
2010*	Pasay City (NCR)	296		1	0.34	DoH 2011 [36]
2010*	Pasig City (NCR)	373		1	0.27	DoH 2011 [36]
2010*	Pateros (NCR)	25		0		DoH 2011 [36]
2010*	Quezon City (NCR)	2569		2	0.82	DoH 2011 [36]
2010*	San Juan City (NCR)	151		0		DoH 2011 [36]
2010*	Taguig City (NCR)	275		2	0.73	DoH 2011 [36]
2010*	Valenzuela City (NCR)	740		6	0.81	DoH 2011 [36]
2011*	NCR	15,427		93	0.60	DoH 2011 [36]
2011*	CAR	1940		9	0.46	DoH 2011 [36]
2011*	Region I	9494		58	0.61	DoH 2011 [36]
2011*	Region II	4773		27	0.57	DoH 2011 [36]
2011*	Region III	13,347		33	0.25	DoH 2011 [36]
2011*	Region IV-A	10,215		56	0.55	DoH 2011 [36]
2011*	Region IV-B	1072		4	0.37	DoH 2011 [36]
2011*	Region V	532		7	1.32	DoH 2011 [36]
2011*	Region VI	2281		28	1.23	DoH 2011 [36]
2011*	Region VII	3264		19	0.58	DoH 2011 [36]
2011*	Region VIII	564		4	0.71	DoH 2011 [36]

Year	Region	DF		DF mortality		Source of data. First author, year [Ref]
		n	Incidence (per 100,000 population)	n	CFR (%)	
2011*	Region IX	836		12	1.44	DoH 2011 [36]
2011*	Region X	1293		4	0.31	DoH 2011 [36]
2011*	Region XI	2509		18	0.72	DoH 2011 [36]
2011*	Region XII	1764		16	0.91	DoH 2011 [36]
2011*	Region XIII	753		4	0.53	DoH 2011 [36]
2011*	ARMM	140		4	2.86	DoH 2011 [36]
2011*	Kalookan City (NCR)	1941		11	0.57	DoH 2011 [36]
2011*	Las Pinas City (NCR)	406		2	0.49	DoH 2011 [36]
2011*	Makati City (NCR)	317		4	1.26	DoH 2011 [36]
2011*	Malabon City (NCR)	795		3	0.38	DoH 2011 [36]
2011*	Mandaluyong City (NCR)	320		1	0.31	DoH 2011 [36]
2011*	Manila City (NCR)	1944		7	0.36	DoH 2011 [36]
2011*	Marikina City (NCR)	377		1	0.27	DoH 2011 [36]
2011*	Muntinlupa City (NCR)	397		2	0.5	DoH 2011 [36]
2011*	Navotas City (NCR)	497		3	0.6	DoH 2011 [36]
2011*	Paranaque City (NCR)	671		3	0.45	DoH 2011 [36]
2011*	Pasay City (NCR)	378		0		DoH 2011 [36]
2011*	Pasig City (NCR)	838		4	0.48	DoH 2011 [36]
2011*	Pateros (NCR)	103		0		DoH 2011 [36]
2011*	Quezon City (NCR)	4611		32	0.69	DoH 2011 [36]
2011*	San Juan City (NCR)	208		0		DoH 2011 [36]
2011*	Taguig City (NCR)	534		6	1.12	DoH 2011 [36]
2011*	Valenzuela City (NCR)	1090		14	1.28	DoH 2011 [36]
2011†	Calabarzon: Cavite province (Region IV-A)	929		5	0.54	NDRRMC 2011 [39]
2011†	Calabarzon: Laguna province (Region IV-A)	1666		2	0.12	NDRRMC 2011 [39]

Year	Region	DF		DF mortality		Source of data. First author, year [Ref]
		n	Incidence (per 100,000 population)	n	CFR (%)	
2011†	Calabarzon: Batangas province (Region IV-A)	1621		11	0.68	NDRRMC 2011 [39]
2011†	Calabarzon: Rizal province (Region IV-A)	1777		9	0.51	NDRRMC 2011 [39]
2011†	Calabarzon: Quezon province (Region IV-A)	785		6	0.76	NDRRMC 2011 [39]
**(B) Local outbreak data from dengue disease Fellows' reports**						
2001 (April–July)	Benguet, CAR	165				DoH 2012 [23]
2001 (22 May–22 June)	San Miguel, Bulacan (Region III)	7		0		DoH 2012 [23]
2003 (26 April–30 June)	Wao, Lanao del Sur (ARMM)	80		2	2	DoH 2012 [23]
2003 (8 June–22 July)	Alfonso XII, Quezon, Palawan (Region IV)	21				DoH 2012 [23]
2010 (Jan–Sept) (11 July–21 Aug)‡	Zamboanga City (Region IX)	2122		22	1.04	DoH 2012 [23]
2010 (Jan–July)	Digos City, Davao del Sur (Region XI)	367		9	2.5	DoH 2012 [23]
2011 (Feb–July)	Batanes province (Region II)	701		1	0.14	DoH 2012 [23]
**(C) Local outbreak data from Regional Surveillance Units**						
2000 (Jan)	Baguio City (CAR)	2		0		DoH 2012 [23]
2000 (Apr–Jul)	Munoz, Nueva Ecija (Region III)	17				DoH 2012 [23]
2000 (May–Aug)	Gen Tinio Nueva Ecija (Region III)	37		0		DoH 2012 [23]
2000 (June–Aug)	Gapan, Nueva Ecija (Region III)	34			2–6	DoH 2012 [23]
2000 (Nov–Dec)	Cantilan, Surigao del Sur (Region XI)	9				DoH 2012 [23]
2001 (Jan–30 June)	Ifugao (CAR)	44		0		DoH 2012 [23]
2001 (June–31 July)	Abra (CAR)	61		0		DoH 2012 [23]

Year	Region	DF		DF mortality		Source of data. First author, year [Ref]
		n	Incidence (per 100,000 population)	n	CFR (%)	
2001 (July 2001)	Batangas Regional Hospital (Region IV)	41				DoH 2012 [23]
2002 (Jan–31 March)	Zamboanga City (Region IX)	75				DoH 2012 [23]
2002 (20 Apr–8 May)	Loakan, Baguio City (CAR)	9		0		DoH 2012 [23]
2002 (2–19 June)	Balud, Masbate (Region V)	24				DoH 2012 [23]
2002 (June–July	Cebu (Region VII)	13			2–15	DoH 2012 [23]
2002 (1 June–6 Aug)	Mountain province (CAR)	56				DoH 2012 [23]
2003 (Jan–5 Sept)	Valenzuela City (NCR	226		3	1.3	DoH 2012 [23]
2003 (3 Sept–5 Oct)	Mambajao, Camiguin (Region X)	13		1	8	DoH 2012 [23]
2004 (Jan–8 Mar)	Guiuan, Eastern Samar (Region VII)	34				DoH 2012 [23]
2004 (Jan–9 July)	Bislig, Butuan City (Region X)	78		6	7.6	DoH 2012 [23]
2004 (May)	La Trinidad, Benguet (CAR)	52		0		DoH 2012 [23]
2005 (May–July)	Pag-asa, Quezon City (NCR)	42		5	12	DoH 2012 [23]
2006 (5 Oct–24 Nov)	Cabuyao, Laguna (Region IV)	38		4	11	DoH 2012 [23]
2007 (Jan–23 June)	Hinobaan, Negros Occidental (Region VI)	68		3	4.4	DoH 2012 [23]
2007 (Jan–5 July)	Sipalay, Negros Occidental (Region VI)	145		0		DoH 2012 [23]
2007 (Jan–July)	Don Carlos, Bukidnon (Region X)	142		3	2.11	DoH 2012 [23]
2007 (Jan–Oct)	Surigao City (Region X)	164		3	1.8	DoH 2012 [23]

Year	Region	DF		DF mortality		Source of data. First author, year [Ref]
		n	Incidence (per 100,000 population)	n	CFR (%)	
2007 (15 May–June)	San Agustin, Iba, Zambales (Region III)	24		1	4.2	DoH 2012 [23]
2007 (June)	Matnog, Sorsogon (Region V)	25		1	4	DoH 2012 [23]
2007 (4 July–30 July)	Northern Samar (Region VII)	562		3	0.5	DoH 2012 [23]
2007 (Jul–8 Aug)	General Santos City (Region XI)	115		5	4	DoH 2012 [23]
2008 (Jan–28 July)	Calasiao, Pangasinan (Region I)	56		1	1.8	DoH 2012 [23]
2008 (Mar–5 July)	Paracelis, Mt. Province (CAR)	56		2	3.6	DoH 2012 [23]
2008 (1 July–4 Aug)	San Mateo, Rizal (Region IV)	17		2	12	DoH 2012 [23]
2009 (Jan–5 Sept)	Tabuk, Kalinga (CAR)	400		3	0.8	DoH 2012 [23]
2009 (Jan–Sept)	Lagawe, Ifugao (CAR)	171		2	1.2	DoH 2012 [23]
2010 (4 Jan–11 June)	Banisilan, North Cotabato (Region XII)	137		4	2.9	DoH 2012 [23]

ARMM, Autonomous Region in Muslim Mindanao; CAR, Cordillera Administrative Region; CFR, case fatality rate; DF, dengue fever; DoH, Department of Health; FHSIS, Field Health Service Information System; NCR, National Capital Region; NDRRMC, National Disaster Risk Reduction and Management Council.

*1 January to 10 September, 2010 or 2011.
†January to August, 2011.
‡Ongoing outbreak
