# Supplementary material for: Epidemiology of Dengue Disease in the Philippines (2000–2011): A Systematic Literature Review
Source: PLoS Negl Trop Dis. 2014 Nov 6;8(11):e3027. doi: 10.1371/journal.pntd.0003027 (PMC4222740; doi:10.1371/journal.pntd.0003027)
Supplement: Table S5 — Age-specific patterns of dengue disease in the Philippines [25], [29] . Population data from: http://www.doh.gov.ph/kp/statistics/demography1.html. Empty cells: data not provided. DF, dengue fever; DHF, dengue haemorrhagic fever; DoH, Department of Health; FHSIS, Field Health Service Information System; NSCB, National Statistical Coordination Board. *http://www.nscb.gov.ph/secstat/d_popnProj.asp. (PDF) [file pntd.0003027.s005.pdf]

Table S5. Age-specific patterns of dengue disease in the Philippines [25, 29].

	<1 year	1–4 years	0–4 years	5–14 years	15–49 years	50–64 years	≥65 years	Unknown	Total
**DF/DHF cases, n (FHSIS) [29]**									
2000	196	1198	1394	3349	1779	90	2		6614
2001	649	4188	4837	10,219	5980	642	247		21,925
2002	467	2594	3061	5895	3502	258	39		12,755
2003	114	644	758	2056	1774	112	50		4750
2004									
2005	138	684	822	1893	1220	135	22		4092
2006	1923	4445	6368	4088	3038	479	337		14,310
2007	355	1892	2247	5430	3945	214	79		11,915
2008	547	2443	2990	5286	4320	286	132		13,014
2009	124	864	988	2623	1913	68	25		5617
2010									
**Mortality cases, n (DoH) [25]**									
2000									
2001									

	**<1 year**	**1–4 years**	**0–4 years**	**5–14 years**	**15–49 years**	**50–64 years**	**≥65 years**	**Unknown**	**Total**
2002									
2003	47	231	278	364	154	14	21		1109
2004	38	183	221	363	147	16	13	1	982
2005	33	209	242	439	162	23	21		1129
2006									
2007									
2008									
2009									
2010									
**Population, n (DoH and NSCB*)**									
2000*			9,902,800	18,643,400	39,256,800	6,196,100	2,947,400		76,946,500
2001									
2002									
2003			9,633,446	18,463,076	42,245,087	7,295,749	3,416,946		81,054,304
2004			9,646,113	18,616,264	43,212,678	7,574,917	3,586,589		82,636,561
2005			9,658,747	18,769,032	44,231,550	7,853,322	3,728,690		84,241,341

	<1 year	1–4 years	0–4 years	5–14 years	15–49 years	50–64 years	≥65 years	Unknown	Total
2006	2,108,280	8,523,823	10,632,103	19,869,459	45,288,309	7,620,687	3,551,952		86,962,510
2007									
2008									
2009									
2010*			10,984,800	20,171,800	49,507,100	9,290,200	4,059,300		94,013,200
**Incidence per 100,000 population (calculated)**									
2000			14.08	17.96	4.53	1.45	0.07		8.60
2001									
2002									
2003			7.87	11.14	4.20	1.54	1.46		5.86
2004									
2005			8.51	10.09	2.76	1.72	0.59		4.86
2006	91.21	52.15	59.89	20.57	6.71	6.29	9.49		16.46
2007									
2008									
2009									

	<1 year	1–4 years	0–4 years	5–14 years	15–49 years	50–64 years	≥65 years	Unknown	Total
2010									
**Case fatality rate (calculated)**									
2000									
2001									
2002									
2003	0.41	0.36	0.37	0.18	0.09	0.13	0.42		0.23
2004									
2005	0.24	0.31	0.29	0.23	0.13	0.17	0.95		0.28
2006									
2007									
2008									
2009									
2010									

Population data from: http://www.doh.gov.ph/kp/statistics/demography1.html. Empty cells: data not provided.

DF, dengue fever; DHF, dengue haemorrhagic fever; DoH, Department of Health; FHSIS, Field Health Service Information System; NSCB, National Statistical Coordination Board. *http://www.nscb.gov.ph/secstat/d_popnProj.asp.
